# Subthalamic nucleus but not entopeduncular nucleus deep brain stimulation enhances neurogenesis in the SVZ-olfactory bulb system of Parkinsonian rats

**DOI:** 10.3389/fncel.2024.1396780

**Published:** 2024-04-30

**Authors:** Mareike Fauser, Jan Philipp Payonk, Hanna Weber, Meike Statz, Christine Winter, Ravit Hadar, Revathi Appali, Ursula van Rienen, Moritz D. Brandt, Alexander Storch

**Affiliations:** ^1^Department of Neurology, University of Rostock, Rostock, Germany; ^2^Institute of General Electrical Engineering, University of Rostock, Rostock, Germany; ^3^Department of Psychiatry and Neurosciences, Charité University Medicine Berlin, Berlin, Germany; ^4^Department of Ageing of Individuals and Society, University of Rostock, Rostock, Germany; ^5^Department of Life, Light and Matter, University of Rostock, Rostock, Germany; ^6^Department of Neurology, University Hospital Carl Gustav Carus Dresden, Dresden, Germany; ^7^German Center for Neurodegenerative Diseases (DZNE) Dresden, Dresden, Germany; ^8^German Center for Neurodegenerative Diseases (DZNE) Rostock/Greifswald, Rostock, Germany

**Keywords:** deep brain stimulation, subthalamic nucleus, entopeduncular nucleus, Parkinson’s disease, 6-hydroxydopamine, adult neurogenesis, electric field distribution

## Abstract

**Introduction:**

Deep brain stimulation (DBS) is a highly effective treatment option in Parkinson’s disease. However, the underlying mechanisms of action, particularly effects on neuronal plasticity, remain enigmatic. Adult neurogenesis in the subventricular zone-olfactory bulb (SVZ-OB) axis and in the dentate gyrus (DG) has been linked to various non-motor symptoms in PD, e.g., memory deficits and olfactory dysfunction. Since DBS affects several of these non-motor symptoms, we analyzed the effects of DBS in the subthalamic nucleus (STN) and the entopeduncular nucleus (EPN) on neurogenesis in 6-hydroxydopamine (6-OHDA)-lesioned hemiparkinsonian rats.

**Methods:**

In our study, we applied five weeks of continuous bilateral STN-DBS or EPN-DBS in 6-OHDA-lesioned rats with stable dopaminergic deficits compared to 6-OHDA-lesioned rats with corresponding sham stimulation. We injected two thymidine analogs to quantify newborn neurons early after DBS onset and three weeks later. Immunohistochemistry identified newborn cells co-labeled with NeuN, TH and GABA within the OB and DG. As a putative mechanism, we simulated the electric field distribution depending on the stimulation site to analyze direct electric effects on neural stem cell proliferation.

**Results:**

STN-DBS persistently increased the number of newborn dopaminergic and GABAergic neurons in the OB but not in the DG, while EPN-DBS does not impact neurogenesis. These effects do not seem to be mediated via direct electric stimulation of neural stem/progenitor cells within the neurogenic niches.

**Discussion:**

Our data support target-specific effects of STN-DBS on adult neurogenesis, a putative modulator of non-motor symptoms in Parkinson’s disease.

## Introduction

Deep brain stimulation (DBS) is a highly effective treatment option in middle- to late-stage Parkinson’s disease (PD), offering long-lasting motor symptom benefits (Deuschl et al., 2013). While motor symptoms improve shortly after DBS onset, new data suggest that there is also a more delayed alleviation of various non-motor symptoms [NMS; (Wolz et al., 2012; Dafsari et al., 2016, 2018)]. The mechanisms behind NMS improvements remain unresolved so far, though an association with location of active electrode contacts has been proposed (Funkiewiez et al., 2003; Petry-Schmelzer et al., 2019). Some NMS, e.g., olfactory dysfunction and depression, can frequently occur as prodromal symptoms (Tolosa et al., 2009) and have been linked to alterations in adult neurogenesis in PD: while for hyposmia, an increase in dopaminergic OB interneurons has been proposed as a pathophysiological mechanisms (Huisman et al., 2004; Mundiñano et al., 2011), hippocampal atrophy in PD has been linked to PD-associated depression and cognitive impairment (Brück et al., 2004; Györfi et al., 2017). Interestingly, according to Braak et al. (2003), the olfactory system is also one of the first regions to be affected by PD neuropathology.

Adult neurogenesis is well-characterized in the neurogenic niches of the subventricular zone (SVZ) of the lateral ventricles and in the dentate gyrus (DG). SVZ-derived adult neural stem/progenitor cells (named herein aNSCs) migrate along the rostral migratory system (RMS) into the olfactory bulb (OB) to differentiate into various interneurons, which play a role in olfactory learning, e.g., odor discrimination (Doetsch et al., 1997; Doetsch, 2003; Breton-Provencher et al., 2009). In contrast, DG-aNSCs contribute to resident neuronal turnover (Jhaveri et al., 2010) and are involved in memory formation and mood regulation (van Praag et al., 1999; Kempermann et al., 2003; Snyder et al., 2005).

In the context of electric stimulation, directed migration of SVZ-aNSCs into the OB is a prerequisite for adequate neuronal turnover and is partly controlled by endogenous electric fields, both in the adult brain and during development (Hotary and Robinson, 1992; Cao et al., 2013, 2015). Experimental direct current electric fields induce cathodal migration without altering aNSC differentiation *in vivo*, with similar findings after biphasic pulsed stimulation (Iwasa et al., 2019; Sefton et al., 2020). *In vitro*, various studies demonstrated influences on migration, proliferation and differentiation of aNSCs, though stimulation paradigms, e.g., regarding field strengths and duration of stimulation, vary greatly (Li et al., 2008; Ariza et al., 2010; Babona-Pilipos et al., 2015; Iwasa et al., 2019).

In PD, impairment of adult neurogenesis has been described both in different animal models and in patients: in the 6-OHDA and the 1-methyl-4-phenyl-1,2,3,6-tetrahydropyridine (MPTP)-lesioned models, dopaminergic denervation resulted in decreased aNSC proliferation and reduced numbers of mature newborn neurons in the respective target regions in some studies; however, more recent studies challenged these findings with either no or even contrary effects of dopamine depletion on neurogenesis (Höglinger et al., 2004; Aponso et al., 2008; Winner et al., 2009; Ermine et al., 2018). In genetic animal models, e.g., LRRK2 G2019S or mutant and wild-type α-synuclein mice, neurogenesis was overall impaired, though congenital dopamine depletion in Pitx3-mutant mice affected NSC proliferation exclusively in younger animals (Winner et al., 2008, 2011; Marxreiter et al., 2009; Kohl et al., 2016; Brandt et al., 2017). Similar findings were obtained in clinical studies: while Höglinger et al. (2004) described reduced aNSC proliferation in the SVZ in patients’ *post-mortem* brains, these findings have been challenged in subsequent investigations (van den Berge et al., 2011; Terreros-Roncal et al., 2021).

The impact of chronic DBS on adult neurogenesis has not been investigated in detail so far: Khaindrava et al. (2011) report unaltered SVZ- and DG-aNSC proliferation after eight days of STN-DBS, but increased survival of newborn neuroblasts and neurons in the 6-OHDA rat model. Other studies in healthy animals or in different psychiatric disease models, e.g., for depression and dementia, mostly report an increase in adult-generated hippocampal neurons, independent of the stimulation site (e.g., entorhinal cortex, anteromedial thalamus) or DBS duration (Toda et al., 2008; Stone et al., 2011; Chamaa et al., 2021; Zhou et al., 2022). The only study on aNSC proliferation in PD patients demonstrated an increase in SVZ precursor cell proliferation in DBS-treated patients compared to both unstimulated PD subjects and healthy controls, though without data on hippocampal neurogenesis (Vedam-Mai et al., 2014).

In the present study, we used the 6-OHDA PD rat model to comparatively investigate the influence of long-term continuous DBS in the STN or the entopeduncular nucleus (EPN; rodent homologue of the human internal globus pallidus) on proliferation and differentiation of aNSCs in the SVZ and the DG as a putative mediator of NMS improvement after DBS. To elucidate direct electrical stimulation of aNSCs as a putative mechanism of DBS action on neurogenesis, we not only compare STN-DBS and EPN-DBS but also simulate the volume of tissue activated (VTA) and the corresponding strength of the electric field within the neurogenic regions.

## Materials and methods

Animals: All procedures were permitted by responsible authorities (Landesdirektion Sachsen, Germany; reference numbers DD24-5131/207/3) and carried out in line with ARRIVE guidelines and the EU Directive 2010/63/EU for animal experiments as reported previously (Fauser et al., 2021). We used female Wistar rats (∼240–260 g at purchase, Charles River Laboratories, Sulzfeld, Germany) that were kept under a 12 h/12 h light-dark cycle and had *ad libitum* access to food and water. Rats were housed 2–3/cage until successful lesioning and singularised after electrode implantation to prevent reciprocal electrode and stimulator destruction. Influences of STN-DBS and EPN-DBS on midbrain dopaminergic systems have already been published from the same cohort (Fauser et al., 2021). Induction of the dopaminergic deficit and DBS surgery were carried out as described before (Fauser et al., 2021) and are detailed in the Supplementary Methods Section. We applied bilateral DBS or respective sham stimulation in all animals and used the contralateral, non-lesioned hemispheres as a healthy control.

Thymidine analogs labeling: All animals were injected intraperitoneally (i.p.) with 57.5 mg/kg BW 5′-iodo-2′-deoxyuridine (IdU; 23 mg/ml in 0.9% NaCl with 0.2 N NaOH; MP Biochemicals, CA, USA) every 12 h for a total of 72 h two days after DBS onset; 21 days later, rats received 42.5 mg/kg BW 5′-chloro-2′-deoxyuridine (CldU; 17 mg/ml in 0.9% NaCl; Sigma-Aldrich, Dorset, UK) i.p. with an identical protocol to label proliferating cells in the respective neurogenic regions (Vega and Peterson, 2005).

Immunohistochemistry: For triple immunostaining of every 6th 40 μm section, we used standard protocols as described before [(Hermann et al., 2009), see Supplementary Methods for details] and the following primary antibodies: rat anti-BrdU for detection of CldU incorporation (1:500; RRID:AB_609568; Bio-Rad Laboratories, CA, USA), mouse anti-BrdU for detection of IdU (1:500; RRID:AB_400326; BD Biosciences, Heidelberg, Germany), rabbit anti-TH (1:500; RRID:AB_390204; Chemicon GmbH, Limburg an der Lahn, Germany), chicken anti-NeuN (1:1000; RRID:AB_11155058; Abcam, Berlin, Germany), rabbit anti-GABA (1:1000; RRID:AB_477652; Sigma-Aldrich, Taufkirchen, Germany), goat anti-DCX (1:100; RRID:AB_2088494; Santa Cruz Biotechnology, Heidelberg, Germany), mouse anti-PH3 (1:100; RRID:AB_331748; Cell Signalling Technologies, Danvers, MA, USA). In a previous study, Vega and Peterson demonstrated that one of the BrdU antibodies used in the present experiments (Bio-Rad Laboratories, CA, USA) specifically detects CldU, while the other BrdU antibody (BD Biosciences, Heidelberg, Germany) selectively binds to IdU (Vega and Peterson, 2005).

The following day, sections were incubated in appropriate secondary antibodies; cell nuclei were counterstained with bisbenzimide H33342 fluorochrome trihydrochloride (Invitrogen, CA, USA) or 4′, 6-diamidino-2-phenylindole dihydrochloride (DAPI; Sigma-Aldrich, Taufkirchen, Germany).

Imaging and quantifications: Respective OB, SVZ and DG sections were imaged and quantified using a Zeiss Laser scanning confocal LSM 700 or a motorized Axio.Observer.Z1 and ZEN Blue software with Tiles and Position Module (Carl Zeiss, Oberkochen, Germany). We chose standardized settings at the beginning of the experiments, which remained unchanged for all subsequent imaging studies. For all quantifications, the rater was blinded to group allocation of the respective slices. For quantitative histology within the OB, we defined regions of interest (ROIs) and placed counting squares with a set volume of 250 × 250 × 40 μm^3^ randomly within the glomerular layer (six ROIs/slice) and granule cell layer (two ROIs/slice) of the OB. These counting squares were again placed in a blinded manner by an employee who was not otherwise part of the study. Within the ROIs in the granule cell layer, we quantified CldU^+^GABA^+^ and IdU^+^GABA^+^ cells. Within the ROIs in the glomerular layer, we analyzed CldU^+^TH^+^, IdU^+^TH^+^, CldU^+^GABA^+^, IdU^+^GABA^+^ cells. CldU^+^IdU^+^ cells were extremely rare. Therefore, they were not quantified. Due to comparatively low numbers of labeled proliferating cells, we quantified IdU^+^, CldU^+^, IdU^+^NeuN^+^ and CldU^+^NeuN^+^ cells within the entire DG. For the SVZ-RMS continuum, we defined counting squares (CS) of 300 μm^3^ × 100 μm^3^ × 40 μm^3^ and placed them in the most dorsal parts of the SVZ within the regions of the highest proliferative activity along the dorsal-ventral axis. Within the CSs, we quantified PH3^+^ proliferating cells and performed intensity measurements using ZEN Blue 2.3 software to quantify densely packed DCX^+^ cells. Coronal slices from Bregma −0.48 mm to < 2.52 mm were defined as SVZ, while slices ≥ 2.52 mm were defined as RMS [according to Paxinos and Watson (2007) and Golmohammadi et al. (2008)].

*In silico* modeling of the electric fields: To adequately characterize the electric field distribution and the volume of tissue activated (VTA) in the rat brain, we used the second version of our open-source simulation platform OSS-DBS (Butenko et al., 2020). For precise modeling of the computational domain, we employed a segmented magnetic resonance image (MRI) from the multidimensional magnetic resonance histology atlas (Johnson et al., 2012). To account for the anisotropic tissue properties of the brain, we use diffusion tensor data estimated in the same space. The dielectric properties of brain tissue, including gray matter, white matter, and cerebrospinal fluid, are estimated based on a the four-dispersion Cole-Cole model (Gabriel et al., 1996). Due to the absence of magnetic induction and the relatively low frequencies, the quasi-static approximation of Maxwell’s equation is valid (van Rienen, 2001). We solved the equation using the finite element method to estimate the potential and the electric field. Thresholding was used to determine the extent of the VTAs. Due to the smaller diameter of axons in rats compared to humans, a relatively high threshold is required to activate them. We chose a threshold of 0.301 V/mm based on our stimulation amplitude and the values proposed by Astrom et al. (2015) for the relationship between electric field distribution and neural activation.

Statistics analyses were carried out with SPSS (version 27, IBM, NY, USA), while data plots and figures were created with GraphPadPrism 9.4.1 (GraphPad Software, CA, USA) and BioRender.com (BioRender, Canada). Shapiro-Wilk test and visual inspection of box plots were applied to test for normal distribution and homogeneity of variances was analyzed with Levene test. Since it cannot be excluded that incorporation of IdU and CldU is variable between the two substances, we did not perform a within-subject analysis for the two injection timepoints, but used *t*-tests or Mann–Whitney-U-tests for group comparisons for each timepoint separately depending on the normal distribution of our data. All data are presented as boxplots with a central mark at the median, bottom, and top edges of the boxes at 25th and 75th percentiles, respectively, and whiskers at the minimum/maximum (dots represent individual values), except data on cell counts of the SVZ-RMS continuum which are presented as mean ± SEM for clarity.

## Results

### Chronic dopaminergic deficiency does not persistently alter adult neurogenesis in the 6-OHDA model

First, we analyzed the effects of chronic dopaminergic deficiency in the 6-OHDA model on net neurogenesis as indicated by thymidine analogs labeling in severely lesioned animals with a stable dopaminergic deficit in closely matched groups: the overall decrease in nigral dopaminergic neurons as well as dopaminergic fiber density in the striatum both dropped by ∼90% [published in Figures 1e, f in Fauser et al. (2021)]. In contrast to previous studies, we investigated neurogenesis in animals with a completed dopaminergic deficit and not during the ongoing degeneration shortly after lesioning (Höglinger et al., 2004; Winner et al., 2009). In the present study, there was no persistent effect 15 weeks after induction of the severe dopaminergic deficiency on aNSC proliferation, when comparing the ipsilateral 6-OHDA-lesioned side with the contralateral non-lesioned hemisphere in the OB and DG (see Supplementary Table 1 for detailed statistics). These findings align with clinical studies in PD patients, in which recent studies report unaltered NSC proliferation compared to healthy, age-matched controls (van den Berge et al., 2011; Terreros-Roncal et al., 2021).

### Chronic STN-DBS persistently increases SVZ-OB neurogenesis in an animal model of stable dopaminergic deficiency

**FIGURE 1:**
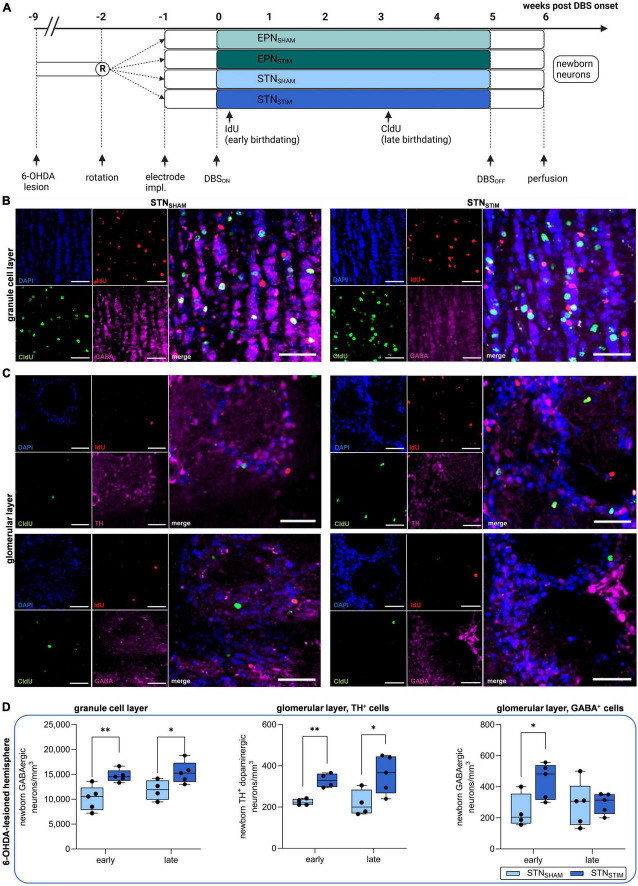
Effects of deep brain stimulation in the subthalamic nucleus (STN-DBS) on adult neurogenesis in the olfactory bulb (OB) in hemiparkinsonian rats. **(A)** Schematic representation of experimental paradigm to investigate the effects of STN-DBS vs. DBS in the entopeduncular nucleus (EPN) on adult neurogenesis in the OB and dentate gyrus (DG) in 6-hydroxydopamine (6-OHDA) hemiparkinsonian rats. To secure a stable dopaminergic deficit, bilateral DBS electrodes were implanted eight weeks after initial 6-OHDA lesion either into the EPN or into the STN followed by high frequency stimulation for five weeks with perfusion of animals one week after termination of stimulation. SHAM animals (electrode implantation without active stimulation) served as controls. IdU and CldU were injected six times bi-daily starting two days after DBS onset and three weeks later. Created with BioRender.com. **(B,C)** Representative immunohistological images of newborn neurons in the OB in STN_SHAM_ and STN_STIM_ conditions. IdU (red) labels newborn neurons that were generated early (two days) after DBS initiation, while CldU (green) indicates newborn neurons after three weeks of continuous DBS. GABAergic neurons (pink) are found both in granule cell layers **(B)** and in glomerular layers (**C**, lower panel), while TH^+^ dopaminergic neurons (pink) are restricted the latter (**C**, upper panel). Cell nuclei were counterstained with DAPI. Scale bar, 50 μm. **(D)** A significant increase in the numbers of newborn GABAergic neurons in the granule cell layer and TH^+^ dopaminergic neurons in the glomerular layer were found both early after STN-DBS onset and three weeks later, while GABAergic neurons in the glomerular layer were only significantly increased immediately after STN-DBS induction compared to sham stimulation. All results were obtained from 6-OHDA-lesioned hemispheres. Data are presented as boxplots with a central mark at the median, bottom, and top edges of the boxes at 25th and 75th percentiles, respectively, and whiskers at the minimum/maximum (dots represent individual values). *represents *P* < 0.05 and **represents *P* < 0.01 from Mann–Whitney-U-tests. 6-OHDA, 6-hydroxydopamine; DBS, deep brain stimulation; STN, subthalamic nucleus; EPN, entopeduncular nucleus; OB, olfactory bulb; IdU, 5-iodo-2′-deoxyuridine; CldU, 5-chloro-2′-deoxyuridine; GABA, gamma aminobutyric acid; TH, tyrosine hydroxylase; DAPI, 4′,6-diamidino-2-phenyl-indol-dihydrochloride.

To assess potential effects of DBS on cellular plasticity within the neurogenic niche of the SVZ-RMS-OB axis, we initially quantified different subtypes of newborn neurons within the glomerular and granule cell layers of the OB from two different time points: early after DBS onset (two days, EARLY) and three weeks later (LATE) with persistent electrical stimulation. Since SVZ-aNSC proliferation seems to be unaffected by DBS (Khaindrava et al., 2011), we here focus on total numbers of terminally differentiated newborn neurons (for details on experimental setups, see Figure 1A).

We analyzed numbers of newborn GABAergic neurons in the granule cell layer as well as dopaminergic (TH^+^) and GABAergic neurons in the glomerular layer in the right, 6-OHDA-lesioned hemisphere (Figures 1B–D). We report significant increases in all three cell types early after DBS onset, though these effects persisted only in the granule cell layer and in dopaminergic, but not GABAergic, cells in the glomerular layer after three weeks. Total numbers of newborn GABAergic neurons in the granule cell layer early after DBS onset in the STN_SHAM_ animals were 10,186 ± 1,106 neurons/mm^3^ and 14,707 ± 552 neurons/mm^3^ in STN_STIM_ animals on the 6-OHDA-lesioned side, while we found 11,869 ± 1,003 and 15,381 ± 987 neurons/mm^3^ in STN_SHAM_ and STN_STIM_ animals after three weeks of continuous STN-DBS (*P* = 0.006 and *P* = 0.04).

In the glomerular cell layer, we quantified two different neuronal subtypes, namely dopaminergic (TH^+^) and GABAergic neurons (Figure 1D): in the lesioned hemisphere, we found a significant increase in both subtypes of newborn neurons after STN-DBS compared to sham stimulation after treatment onset, which was, however, only maintained in dopaminergic neurons. Newborn dopaminergic neurons in the glomerular layer early after DBS onset were 224 ± 8 and 330 ± 17 neurons/mm^3^ in STN_SHAM_ and STN_STIM_ animals, respectively, and 218 ± 31 and 358 ± 41 neurons/mm^3^ under chronic DBS treatment in 6-OHDA-lesioned hemispheres (*P* = 0.004 and *P* = 0.04). Numbers of GABAergic neurons were 241 ± 55 and 439 ± 51 neurons/mm^3^ early after DBS onset in STN_SHAM_ and STN_STIM_ cohorts and 286 ± 64 and 294 ± 30 neurons/mm^3^ during long-term DBS in lesioned hemispheres (*P* = 0.04 and *P* = 0.91; Figure 1D).

In addition, we quantified newborn neurons in contralateral non-lesioned hemispheres. Here, only numbers of newborn dopaminergic neurons in the glomerular cell layer were significantly increased with 286 ± 27 and 524 ± 80 neurons/mm^3^ in STN_SHAM_ and STN_STIM_ animals early after DBS onset and 307 ± 50 and 608 ± 95 neurons/mm^3^ after chronic treatment (*P* = 0.03 and *P* = 0.04, respectively; for complete data see Supplementary Figures 1A–C and Supplementary Table 2).

### An increase in OB dopaminergic neurons in bilateral EPN-DBS is not maintained during chronic stimulation

**FIGURE 2:**
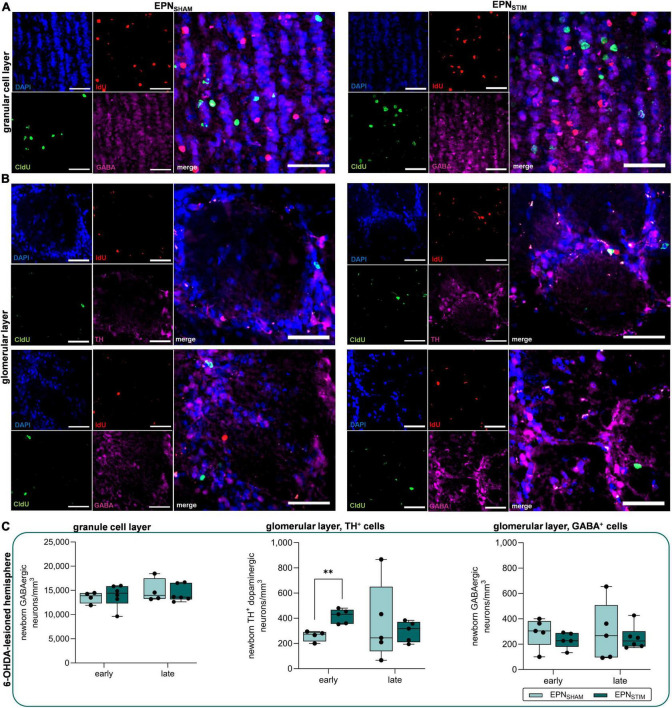
Effects of deep brain stimulation in the entopeduncular nucleus (EPN-DBS) on adult neurogenesis in the olfactory bulb (OB) of hemiparkinsonian rats. **(A,B)** Representative immunohistological images of newborn neurons the OB in EPN_SHAM_ and EPN_STIM_ conditions. IdU (red) labels newborn neurons that were generated early (two days) after DBS onset, while CldU (green) indicates newborn neurons after three weeks of continuous DBS. GABAergic neurons (pink) were found both in the granule cell layer **(A)** and in the glomerular layer (**B**, lower panel), while TH^+^ dopaminergic neurons (pink) were restricted to the latter (**B**, upper panel). Cell nuclei were counterstained with DAPI. Scale bar, 50 μm. **(C)** In 6-OHDA-lesioned hemispheres, bilateral EPN-DBS did not alter the numbers of newborn GABAergic neurons in the granule cell layer or glomerular layer compared to sham stimulation, but increased dopaminergic neuron counts in the glomerular layer early after DBS onset; however, this was not maintained after three weeks. Data are presented as boxplots with a central mark at the median, bottom, and top edges of the boxes at 25th and 75th percentiles, respectively, and whiskers the minimum/maximum (dots represent individual values). **represents *P* < 0.01 from Mann–Whitney-U-tests. DBS, deep brain stimulation; EPN, entopeduncular nucleus; OB, olfactory bulb; IdU, 5-iodo-2′-deoxyuridine; CldU–5-chloro-2′-deoxyuridine; GABA, gamma aminobutyric acid; TH, tyrosine hydroxylase; DAPI–4′,6-diamidino-2-phenyl-indol-dihydrochloride.

Prompted by previous studies on STN- and EPN-DBS (or Gpi-DBS in humans), in which dopaminergic plasticity was restricted to STN-DBS (Fischer et al., 2015; Fauser et al., 2021), we were interested whether the different stimulation targets also have differential effects on adult neurogenesis. Again, we quantified newborn GABAergic and dopaminergic neurons in the granule and glomerular layers of the OB. We only found a significant increase in numbers of newborn dopaminergic neurons in 6-OHDA-lesioned hemispheres in the OB early after DBS onset, which was, however, not maintained during chronic DBS (*P* = 0.003; Figure 2, Supplementary Figure 1D and Supplementary Table 2).

### Chronic STN- and EPN-DBS does not influence hippocampal neurogenesis

Impairment of hippocampal neurogenesis has been reported to be associated with the development of mood disorders and cognitive decline (Winocur et al., 2006; Zhou et al., 2022), two highly prevalent NMS in PD (Rodriguez-Blazquez et al., 2020). In our study, bilateral STN-DBS did not provoke any alterations in the numbers of newborn neurons generated shortly after DBS onset in the subgranular zone (SGZ; *P* = 0.16) or granular zone (GZ; *P* = 0.50) and three weeks later in 6-OHDA lesioned hemispheres (*P* = 0.53 and *P* = 0.58). In addition, STN-DBS did not alter neurogenesis in stimulated, non-lesioned hemispheres compared to sham stimulation. Similar findings were obtained in EPN-DBS (see Supplementary Figure 2 and Supplementary Tables 1, 2 for details).

### STN-DBS does not persistently increase SVZ-aNSC proliferation beyond the stimulation period

Since DBS had to be terminated one week prior to perfusion due to technical limitations, we next assessed SVZ-aNSC proliferation in 6-OHDA-lesioned animals without active DBS. In a previous study, STN-DBS effects persisted beyond the stimulation period by several weeks (Fauser et al., 2021). We quantified proliferating PH3^+^ aNSCs and DCX^+^ neuroblasts in the SVZ and the entire RMS in STN_SHAM_ and STN_STIM_ animals. Animals from EPN groups were not further analyzed. Dense DCX^+^ neuroblasts were quantified using intensity measurements (Supplementary Figures 3A, B). We found no significant differences in the total number of PH3^+^ proliferating aNSCs in non-lesioned and lesioned hemispheres within the entire SVZ (*P* = 0.56 and *P* = 0.11) and RMS (*P* = 0.41 and *P* = 0.91), nor in the density of DCX^+^ neuroblasts between STN_SHAM_ and STN_STIM_ animals in the entire SVZ (both *P* = 0.56) and RMS (*P* = 0.12 and *P* = 0.89; all from Mann–Whitney-U-tests; Supplementary Figures 3C, D), respectively.

### The VTA of either DBS target does not encompass the neurogenic regions in the SVZ or DG, respectively

**FIGURE 3:**
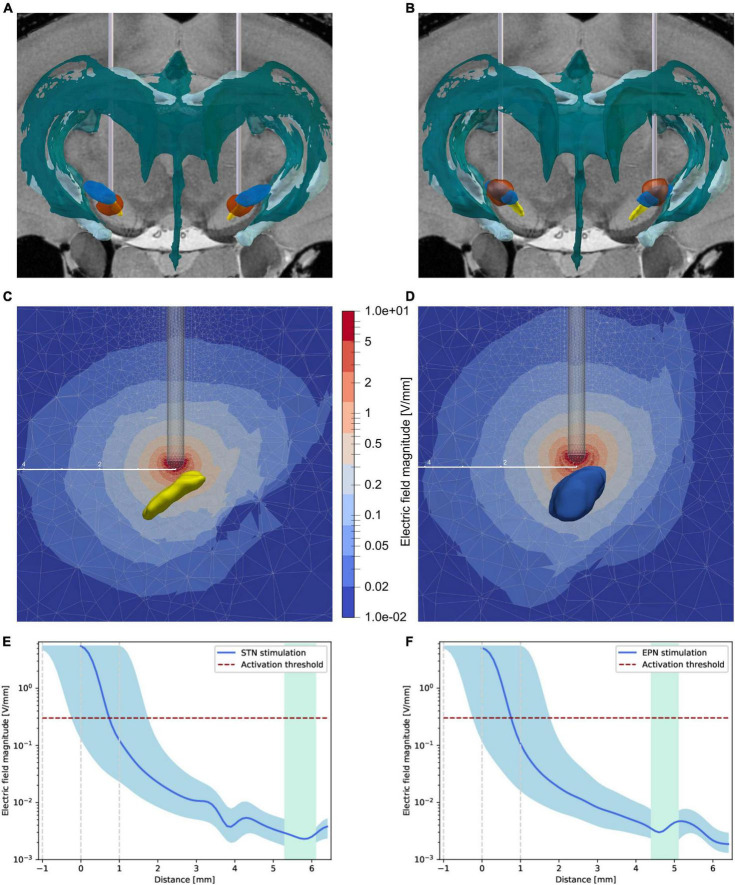
Distribution of the electric fields induced by STN-DBS and EPN-DBS. **(A,B)** Electrode reconstruction within the **(A)** STN (yellow structure) and **(B)** EPN (blue structure) in transversal views illustrating the volume of tissue activated (VTA, circumferential red circle around the electrodes) with respect to the two neurogenic niches, namely the SVZ adjusted to the lateral ventricles of the ventricular system (dark green structure) and the dentate gyrus (bright green structure). To illustrate the distance of the VTA to the neurogenic niches in 3D, please refer to Supplementary Videos 1, 2. **(C,D)** Sectional view of the electric field strength in V/mm during **(C)** STN-DBS and **(D)** EPN-DBS. The electrode position relative to STN (yellow structure) and EPN (blue structure) are shown in transversal sections. The color code represents the electric field magnitude in V/mm. The given distance scale (mm) shows that the electric field strength decreases rapidly. **(E,F)** The electric field strength is estimated over the shortest distance between the electrode tip and the neurogenic region. It is shown in blue for **(E)** STN-DBS and **(F)** EPN-DBS with an error margin (light blue area) representing the electric field strength for an electrode placed ± 1 mm in the direction of the SVZ. The green area spans the neurogenic region of the SVZ that was analyzed here (from bregma –0.48 to 2.52 mm). The horizontal red dashed line indicates the chosen activation threshold based on the work of Astrom et al. (2015). DBS, deep brain stimulation; EPN, entopeduncular nucleus; STN, subthalamic nucleus; VTA, volume of tissue activated.

Since the process of neurogenesis is in part regulated by endogenous electric fields both *in vitro* and *in vivo* (Iwasa et al., 2019; Sefton et al., 2020), we analyzed the actual electric field strengths within the neurogenic region of the SVZ. Volumes of tissue activated (VTAs) were calculated with OSS-DBS, showing the rapid decrease of the electric field strengths around the electrode tip (Figure 3, Supplementary Figure 4 and Supplementary Videos 1, 2). Using a threshold of 0.301 V/mm, as explained in the “Materials and methods” section, the VTAs had variable radii depending on the direction with brain morphology but were clearly below 1.5 mm.

Further, the magnitude of the electric field has been estimated on the shortest path between the stimulation target (electrode tip) and the neurogenic region, which may not show the highest field strengths but clearly showed that the impact of the electric field in the target region is negligibly small (Figures 3E, F). Comparing STN-DBS against EPN-DBS, no differences in the electric field magnitude in the neurogenic regions could be observed.

## Discussion

The present study indicates that bilateral long-term STN-DBS increases forebrain neurogenesis within the SVZ-RMS-OB continuum in a 6-OHDA hemiparkinsonian rodent model of stable dopaminergic deficiency. These neuroplasticity effects are specific for both the stimulation site, i.e., not reproducible with EPN-DBS, and the respective neurogenic niche, as hippocampal neurogenesis was entirely unaltered. In healthy, non-lesioned hemispheres, STN-DBS also enhanced the numbers of newly-generated dopaminergic interneurons in the OB. In addition, we are the first to show that these STN-DBS-mediated alterations in neurogenesis persist during long-term DBS. Since we compared active with sham DBS, subthalamotomy-like effects of electrode placement into the STN do not explain the STN-DBS actions on neurogenesis.

In contrast to previous studies (Höglinger et al., 2004; Winner et al., 2009; Khaindrava et al., 2011), our model of severe, but—at DBS onset—already completed dopaminergic deficiency did not show alterations in aNSC proliferation in the neurogenic niches when compared to non-lesioned hemispheres. The main differences between these studies and ours are the site of lesioning and the interval between neurotoxic lesioning and labeling of proliferating aNSCs. Regarding the time interval, a direct toxic effect of the applied substances on aNSC proliferation in previous studies could be discussed as well as neuroimmunological mechanisms involving microgliosis and reactive astrogliosis, which also resolve over time (Marinova-Mutafchieva et al., 2009; Walsh et al., 2011; Fauser et al., 2021). However, our findings are in line with our previous study in a genetic Pitx3-mutant PD model demonstrating that embryonic degeneration of midbrain dopaminergic neurons affects early postnatal but not late neurogenesis in both the SVZ and hippocampus (Brandt et al., 2017). Furthermore, some human studies describe unaltered aNSC proliferation in PD patients with a long disease duration compared to age-matched controls (van den Berge et al., 2011; Terreros-Roncal et al., 2021). Nevertheless, increased forebrain neuronal turnover might still lead to beneficial effects concerning related NMS, e.g., hyposmia, as it has already been described in a clinical study (Saatçi et al., 2019).

Our study is the first to describe that EPN-DBS does not alter cellular plasticity in any of the neurogenic regions. In line with these findings, we and others already reported a lack of plasticity in the dopaminergic systems after EPN-DBS, which had been repeatedly demonstrated after STN-DBS (Khaindrava et al., 2011; Fischer et al., 2015; Fauser et al., 2021). We hypothesize that mechanisms of action of DBS might differ between stimulation sites, since e.g., DBS in different limbic targets for psychiatric conditions consistently increases hippocampal plasticity, which has not been modulated at all in our study (Toda et al., 2008; Stone et al., 2011; Chamaa et al., 2021). Regarding comparative studies on EPN-(or GPI-DBS in humans) and STN-DBS in PD, the pattern of associated motor and non-motor improvements seems to differ between the stimulation targets (Odekerken et al., 2013; Dafsari et al., 2020), while overall cognitive and psychiatric outcomes are equivalent over time (Boel et al., 2016).

Growing evidence from both *in vitro* and *in vivo* studies demonstrates that aNSCs are electrosensitive cells (Babona-Pilipos et al., 2011; Iwasa et al., 2017; Sefton et al., 2020). However, our *in silico* studies on the electric field strength using DBS parameters used in clinical settings for PD demonstrated that the VTA of both stimulation targets does not include the neurogenic niches. Indeed, the electric field strength acting on the aNSC niches in our studies using clinical DBS parameters is much smaller (< 0.01 V/mm) than those used to study aNSC properties *in vitro* [e.g., 250 mV/mm during the cathodal pulse of asymmetric biphasic stimulation applied to the striatum for only 1 h; (Iwasa et al., 2019; Sefton et al., 2020)]. Of note, a detailed simulation of the electric field strength actually acting on the aNSCs within their niches was not reported in previous studies. However, a direct comparison between our stimulation parameters between these studies is not feasible, due to widely different technical paradigms, e.g., electrodes, stimulation targets and parameters, and the duration of stimulation.

Moreover, according to our simulations of the electric fields and corresponding VTAs, the specific effects of STN-DBS compared to EPN-DBS are not sufficiently explained by differences in local electric field strengths within the respective neurogenic niches. Together, direct electrical stimulation of the neurogenic niche as a mechanism of STN-DBS effects on SVZ-OB neurogenesis is highly unlikely. In contrast, the specificity of the neurogenic effects of STN-DBS with respect to the stimulation target is a strong argument for an indirect stimulation of direct or indirect afferents to the SVZ neurogenic niche as already shown for hippocampal neurogenesis enhanced by electrical stimulation of hippocampal afferents (Derrick et al., 2000; Bruel-Jungerman et al., 2006; Toda et al., 2008; Encinas et al., 2011; Stone et al., 2011). However, the corresponding neurogenic network regulating SVZ-OB neurogenesis (Chen et al., 2023) with regard to STN-DBS effects remains to be determined, especially since direct interconnections between the STN and the medial part of the striatum/SVZ are only sparse (Groenewegen and Berendse, 1990; Schmitt et al., 2016).

In a single previous study on DBS actions on adult neurogenesis in the 6-OHDA model, the authors reported an increased survival of newborn neuroblasts after eight days of STN-DBS within the OB and the DG without concordant alterations in aNSC proliferation rates (Khaindrava et al., 2011). The authors concluded that these neurogenic effects of short-term STN-DBS are not directly linked to dopamine function. In our study, long-term STN-DBS did also not persistently alter aNSC proliferation in the SVZ/RMS. Whether STN-DBS instead acts either on aNSC differentiation or neuroblast survival needs to be elucidated in future studies.

Our study is limited regarding its small animal numbers due to the very high experimental effort in such cohorts; however, the final group sizes are in the range of similar experimental DBS studies (Spieles-Engemann et al., 2011; Fischer et al., 2017; Musacchio et al., 2017). We tried to minimize this limitation by using closely matched groups according to their amphetamine-induced rotational behavior, which sufficiently correlates with between substantia nigra pars compacta (SNpc) dopaminergic cell loss (Tronci et al., 2012). Moreover, we used the contralateral, non-lesioned hemisphere as a control condition and did not include additional healthy or sham-lesioned animals. Therefore, we cannot exclude that neurogenesis might already be altered in these animals due to the contralateral lesion; however, previous studies did not detect differences in contralateral neurogenesis compared to sham-lesioned animals (Höglinger et al., 2004; Suzuki et al., 2010; Fricke et al., 2016). In addition, we included only female animals, limiting our results’ generalizability. Previous studies demonstrated alterations in 6-OHDA susceptibility, as well as influences of sex hormones on adult neurogenesis in female mice compared to their male counterparts and (Tanapat et al., 1999; Somensi et al., 2021). However, our study therefore contributes to a limited number of studies in female animals (Beery and Zucker, 2011), in which influences of sex and sex hormones on adult neurogenesis remain contradictory (Tanapat et al., 1999; Lagace et al., 2007; Yagi and Galea, 2019). In translational terms, the 6-OHDA model does not adequately reflect the neuropathological and clinical hallmarks of human PD, e.g., α-synuclein accumulation and a chronic progressive disease course (Braak et al., 2003). This might limit the transferability of our results into clinical application. However, even most rodent models of synucleinopathies do not present with a similar degree of midbrain dopaminergic degeneration as PD patients (Kordower et al., 2013; Nuber et al., 2013; Polissidis et al., 2021).

It has been previously reported that increased numbers of dopaminergic interneurons in the OB might actually be associated with impaired olfactory function in different neurodegenerative diseases (Huisman et al., 2004; Mundiñano et al., 2011); therefore, our findings of an enhancement in TH^+^ OB neuron counts (in both dopamine-depleted and healthy hemispheres) might even have detrimental effects. Behavioral studies are therefore required to verify the assumption that modulations of neurogenesis might influence certain NMS, but also to better understand DBS influences in healthy subjects, as our results indicate concordant effects, e.g., on the generation of dopaminergic interneurons in the OB, in both healthy and dopamine-depleted conditions. However, such behavioral studies cannot be conducted in a unilateral model of dopaminergic deficiency, as the unaffected hemisphere partly fills in for the unilateral deficit. This has already been shown in behavioral studies on olfaction in 6-OHDA rats, in which no signs of hyposmia could be detected (Ilkiw et al., 2019; Alberts et al., 2022). However, other NMSs, such as anhedonia or depression, are present in unilaterally lesioned animals (Ilkiw et al., 2019). Therefore, we recommend further studies in animals with generalized neuropathology, e.g., transgenic animals with α-synuclein overexpression, but also in healthy control subjects to further dissect the specificity of DBS effects in neurodegenerative diseases.

## Conclusion

Our study provides first evidence that long-term STN-DBS persistently increases SVZ-OB neurogenesis, but not hippocampal neurogenesis in the 6-OHDA PD model of severe, unilateral dopaminergic deficiency. These effects are stimulation-site specific and cannot be reproduced with EPN-DBS, which is, however, equally efficient in treating motor symptoms in PD patients. Further studies to determine behavioral benefits of increased neurogenesis on forebrain-related NMS, e.g., olfaction and hedonistic behavior, need to be carried out, possibly in animal models that more closely resemble the human disease, e.g., models of synucleinopathy. These findings might help to optimize DBS therapy concerning NMS, which often reduce the quality of life to a greater extent than PD motor impairments.

## Data availability statement

The raw data supporting the conclusions of this article will be made available by the authors, without undue reservation.

## Ethics statement

The animal study was approved by the Landesdirektion Sachsen, Germany; reference number DD24-5131/207/3. The study was conducted in accordance with the local legislation and institutional requirements.

## Author contributions

MF: Funding acquisition, Investigation, Methodology, Visualization, Writing – original draft. JP: Methodology, Software, Visualization, Writing – original draft. HW: Investigation, Methodology, Writing – review & editing. MS: Formal analysis, Investigation, Writing – review & editing. CW: Conceptualization, Resources, Writing – review & editing. RH: Methodology, Supervision, Writing – review & editing. RA: Funding acquisition, Methodology, Software, Supervision, Writing – review & editing. UvR: Conceptualization, Funding acquisition, Supervision, Writing – review & editing. MB: Conceptualization, Methodology, Supervision, Writing – review & editing. AS: Conceptualization, Formal analysis, Supervision, Visualization, Writing – review & editing.

## References

[B1] Alberts T. Antipova V. Holzmann C. Hawlitschka A. Schmitt O. Kurth J. (2022). Olfactory bulb D2/D3 receptor availability after intrastriatal botulinum neurotoxin-a injection in a unilateral 6-OHDA rat model of Parkinson’s disease. *Toxins* 14:94. 10.3390/toxins14020094 PMC8879205 35202123

[B2] Aponso P. Faull R. Connor B. (2008). Increased progenitor cell proliferation and astrogenesis in the partial progressive 6-hydroxydopamine model of Parkinson’s disease. *Neuroscience* 151 1142–1153. 10.1016/j.neuroscience.2007.11.036 18201835

[B3] Ariza C. Fleury A. Tormos C. Petruk V. Chawla S. Oh J. (2010). The influence of electric fields on hippocampal neural progenitor cells. *Stem Cell Rev Rep.* 6 585–600. 10.1007/s12015-010-9171-0 20665129

[B4] Astrom M. Diczfalusy E. Martens H. Wardell K. (2015). Relationship between neural activation and electric field distribution during deep brain stimulation. *IEEE Trans. Biomed. Eng.* 62 664–672. 10.1109/TBME.2014.2363494 25350910

[B5] Babona-Pilipos R. Droujinine I. Popovic M. Morshead C. (2011). Adult subependymal neural precursors, but not differentiated cells, undergo rapid cathodal migration in the presence of direct current electric fields. *PLoS One* 6:e23808. 10.1371/journal.pone.0023808 21909360 PMC3166127

[B6] Babona-Pilipos R. Pritchard-Oh A. Popovic M. Morshead C. (2015). Biphasic monopolar electrical stimulation induces rapid and directed galvanotaxis in adult subependymal neural precursors. *Stem Cell Res. Ther.* 6:67. 10.1186/s13287-015-0049-6 25888848 PMC4413998

[B7] Beery A. Zucker I. (2011). Sex bias in neuroscience and biomedical research. *Neurosci. Biobehav. Rev.* 35 565–572. 10.1016/j.neubiorev.2010.07.002 20620164 PMC3008499

[B8] Boel J. Odekerken V. Schmand B. Geurtsen G. Cath D. Figee M. (2016). Cognitive and psychiatric outcome 3 years after globus pallidus pars interna or subthalamic nucleus deep brain stimulation for Parkinson’s disease. *Parkinsonism Relat. Disord.* 33 90–95. 10.1016/j.parkreldis.2016.09.018 27688200

[B9] Braak H. Del Tredici K. Rüb U. de Vos R. Jansen Steur E. Braak E. (2003). Staging of brain pathology related to sporadic Parkinson’s disease. *Neurobiol. Aging* 24 197–211. 10.1016/s0197-4580(02)00065-9 12498954

[B10] Brandt M. Krüger-Gerlach D. Hermann A. Meyer A. Kim K. Storch A. (2017). Early postnatal but not late adult neurogenesis is impaired in the Pitx3-mutant animal model of Parkinson’s Disease. *Front. Neurosci.* 11:471. 10.3389/fnins.2017.00471 28883785 PMC5573808

[B11] Breton-Provencher V. Lemasson M. Peralta M. Saghatelyan A. (2009). Interneurons produced in adulthood are required for the normal functioning of the olfactory bulb network and for the execution of selected olfactory behaviors. *J. Neurosci.* 29 15245–15257. 10.1523/JNEUROSCI.3606-09.2009 19955377 PMC6665973

[B12] Brück A. Kurki T. Kaasinen V. Vahlberg T. Rinne J. (2004). Hippocampal and prefrontal atrophy in patients with early non-demented Parkinson’s disease is related to cognitive impairment. *J. Neurol. Neurosurg. Psychiatry* 75 1467–1469. 10.1136/jnnp.2003.031237 15377698 PMC1738757

[B13] Bruel-Jungerman E. Davis S. Rampon C. Laroche S. (2006). Long-term potentiation enhances neurogenesis in the adult dentate gyrus. *J. Neurosci.* 26 5888–5893. 10.1523/JNEUROSCI.0782-06.2006 16738230 PMC6675234

[B14] Butenko K. Bahls C. Schröder M. Köhling R. van Rienen U. (2020). OSS-DBS: Open-source simulation platform for deep brain stimulation with a comprehensive automated modeling. *PLoS Comput. Biol.* 16:e1008023. 10.1371/journal.pcbi.1008023 32628719 PMC7384674

[B15] Cao L. Pu J. Scott R. Ching J. McCaig C. (2015). Physiological electrical signals promote chain migration of neuroblasts by up-regulating P2Y1 purinergic receptors and enhancing cell adhesion. *Stem Cell Rev. Rep.* 11 75–86. 10.1007/s12015-014-9524-1 25096637 PMC4333314

[B16] Cao L. Wei D. Reid B. Zhao S. Pu J. Pan T. (2013). Endogenous electric currents might guide rostral migration of neuroblasts. *EMBO Rep.* 14 184–190. 10.1038/embor.2012.215 23328740 PMC3596136

[B17] Chamaa F. Darwish B. Nahas Z. Al-Chaer E. Saadé N. Abou-Kheir W. (2021). Long-term stimulation of the anteromedial thalamus increases hippocampal neurogenesis and spatial reference memory in adult rats. *Behav. Brain Res.* 402:113114. 10.1016/j.bbr.2021.113114 33417991

[B18] Chen Y. Ren P. He X. Yan F. Gu R. Bai J. (2023). Olfactory bulb neurogenesis depending on signaling in the subventricular zone. *Cereb. Cortex* 33 11102–11111. 10.1093/cercor/bhad349 37746807

[B19] Dafsari H. Dos Santos Ghilardi M. Visser-Vandewalle V. Rizos A. Ashkan K. Silverdale M. (2020). Beneficial nonmotor effects of subthalamic and pallidal neurostimulation in Parkinson’s disease. *Brain Stimul.* 13 1697–1705. 10.1016/j.brs.2020.09.019 33038595

[B20] Dafsari H. Reddy P. Herchenbach C. Wawro S. Petry-Schmelzer J. Visser-Vandewalle V. (2016). Beneficial effects of bilateral subthalamic stimulation on non-motor symptoms in Parkinson’s Disease. *Brain Stimul.* 9 78–85. 10.1016/j.brs.2015.08.005 26385442

[B21] Dafsari H. Silverdale M. Strack M. Rizos A. Ashkan K. Mahlstedt P. (2018). Nonmotor symptoms evolution during 24 months of bilateral subthalamic stimulation in Parkinson’s disease. *Mov. Disord.* 33 421–430. 10.1002/mds.27283 29465787

[B22] Derrick B. York A. Martinez J. (2000). Increased granule cell neurogenesis in the adult dentate gyrus following mossy fiber stimulation sufficient to induce long-term potentiation. *Brain Res.* 857 300–307. 10.1016/s0006-8993(99)02464-6 10700582

[B23] Deuschl G. Paschen S. Witt K. (2013). Clinical outcome of deep brain stimulation for Parkinson’s disease. *Handb. Clin. Neurol.* 116 107–128. 10.1016/B978-0-444-53497-2.00010-3 24112889

[B24] Doetsch F. (2003). The glial identity of neural stem cells. *Nat. Neurosci.* 6 1127–1134. 10.1038/nn1144 14583753

[B25] Doetsch F. García-Verdugo J. Alvarez-Buylla A. (1997). Cellular composition and three-dimensional organization of the subventricular germinal zone in the adult mammalian brain. *J. Neurosci.* 17 5046–5061. 10.1523/JNEUROSCI.17-13-05046.1997 9185542 PMC6573289

[B26] Encinas J. Hamani C. Lozano A. Enikolopov G. (2011). Neurogenic hippocampal targets of deep brain stimulation. *J. Comp. Neurol.* 519 6–20. 10.1002/cne.22503 21120924 PMC3042399

[B27] Ermine C. Wright J. Frausin S. Kauhausen J. Parish C. Stanic D. (2018). Modelling the dopamine and noradrenergic cell loss that occurs in Parkinson’s disease and the impact on hippocampal neurogenesis. *Hippocampus* 28 327–337. 10.1002/hipo.22835 29431270 PMC5969306

[B28] Fauser M. Ricken M. Markert F. Weis N. Schmitt O. Gimsa J. (2021). Subthalamic nucleus deep brain stimulation induces sustained neurorestoration in the mesolimbic dopaminergic system in a Parkinson’s disease model. *Neurobiol. Dis.* 156:105404. 10.1016/j.nbd.2021.105404 34044146

[B29] Fischer D. Collier T. Cole-Strauss A. Wohlgenant S. Lipton J. Steece-Collier K. (2015). High-frequency stimulation of the rat entopeduncular nucleus does not provide functional or morphological neuroprotection from 6-hydroxydopamine. *PLoS One* 10:e0133957. 10.1371/journal.pone.0133957 26222442 PMC4519335

[B30] Fischer D. Manfredsson F. Kemp C. Cole-Strauss A. Lipton J. Duffy M. (2017). Subthalamic nucleus deep brain stimulation does not modify the functional deficits or axonopathy induced by nigrostriatal α-synuclein overexpression. *Sci. Rep.* 7:16356. 10.1038/s41598-017-16690-x 29180681 PMC5703955

[B31] Fricke I. Viel T. Worlitzer M. Collmann F. Vrachimis A. Faust A. (2016). 6-hydroxydopamine-induced Parkinson’s disease-like degeneration generates acute microgliosis and astrogliosis in the nigrostriatal system but no bioluminescence imaging-detectable alteration in adult neurogenesis. *Eur. J. Neurosci.* 43 1352–1365. 10.1111/ejn.13232 26950181

[B32] Funkiewiez A. Ardouin C. Krack P. Fraix V. Van Blercom N. Xie J. (2003). Acute psychotropic effects of bilateral subthalamic nucleus stimulation and levodopa in Parkinson’s disease. *Mov. Disord.* 18 524–530. 10.1002/mds.10441 12722166

[B33] Gabriel S. Lau R. Gabriel C. (1996). The dielectric properties of biological tissues: III. Parametric models for the dielectric spectrum of tissues. *Phys. Med. Biol.* 41 2271–2293. 10.1088/0031-9155/41/11/003 8938026

[B34] Golmohammadi M. Blackmore D. Large B. Azari H. Esfandiary E. Paxinos G. (2008). Comparative analysis of the frequency and distribution of stem and progenitor cells in the adult mouse brain. *Stem Cells* 26 979–987. 10.1634/stemcells.2007-0919 18203672

[B35] Groenewegen H. Berendse H. (1990). Connections of the subthalamic nucleus with ventral striatopallidal parts of the basal ganglia in the rat. *J. Comp. Neurol.* 294 607–622. 10.1002/cne.902940408 2341628

[B36] Györfi O. Nagy H. Bokor M. Moustafa A. Rosenzweig I. Kelemen O. (2017). Reduced CA2-CA3 hippocampal subfield volume is related to depression and normalized by l-DOPA in newly diagnosed Parkinson’s Disease. *Front. Neurol.* 8:84. 10.3389/fneur.2017.00084 28367136 PMC5355434

[B37] Hermann A. Suess C. Fauser M. Kanzler S. Witt M. Fabel K. (2009). Rostro-caudal gradual loss of cellular diversity within the periventricular regions of the ventricular system. *Stem Cells* 27 928–941. 10.1002/stem.21 19353521

[B38] Höglinger G. Rizk P. Muriel M. Duyckaerts C. Oertel W. Caille I. (2004). Dopamine depletion impairs precursor cell proliferation in Parkinson disease. *Nat. Neurosci.* 7 726–735. 10.1038/nn1265 15195095

[B39] Hotary K. Robinson K. (1992). Evidence of a role for endogenous electrical fields in chick embryo development. *Development* 114 985–996. 10.1242/dev.114.4.985 1618158

[B40] Huisman E. Uylings H. Hoogland P. V. (2004). A 100% increase of dopaminergic cells in the olfactory bulb may explain hyposmia in Parkinson’s disease. *Mov. Disord.* 19 687–692. 10.1002/mds.10713 15197709

[B41] Ilkiw J. Kmita L. Targa A. Noseda A. Rodrigues L. Dorieux F. (2019). Dopaminergic lesion in the olfactory bulb restores olfaction and induces depressive-like behaviors in a 6-OHDA model of Parkinson’s Disease. *Mol. Neurobiol.* 56 1082–1095. 10.1007/s12035-018-1134-5 29869198

[B42] Iwasa S. Babona-Pilipos R. Morshead C. (2017). Environmental factors that influence stem cell migration: An “Electric Field”. *Stem Cells Int.* 2017:4276927. 10.1155/2017/4276927 28588621 PMC5447312

[B43] Iwasa S. Rashidi A. Sefton E. Liu N. Popovic M. Morshead C. (2019). Charge-balanced electrical stimulation can modulate neural precursor cell migration in the presence of endogenous electric fields in mouse brains. *eNeuro* 6:ENEURO.0382-19.2019. 10.1523/ENEURO.0382-19.2019 31772032 PMC6978916

[B44] Jhaveri D. Mackay E. Hamlin A. Marathe S. Nandam L. Vaidya V. (2010). Norepinephrine directly activates adult hippocampal precursors via beta3-adrenergic receptors. *J. Neurosci.* 30 2795–2806. 10.1523/JNEUROSCI.3780-09.2010 20164362 PMC2837927

[B45] Johnson G. Calabrese E. Badea A. Paxinos G. Watson C. (2012). A multidimensional magnetic resonance histology atlas of the Wistar rat brain. *Neuroimage* 62 1848–1856. 10.1016/j.neuroimage.2012.05.041 22634863 PMC3408821

[B46] Kempermann G. Gast D. Kronenberg G. Yamaguchi M. Gage F. (2003). Early determination and long-term persistence of adult-generated new neurons in the hippocampus of mice. *Development* 130 391–399. 10.1242/dev.00203 12466205

[B47] Khaindrava V. Salin P. Melon C. Ugrumov M. Kerkerian-Le-Goff L. Daszuta A. (2011). High frequency stimulation of the subthalamic nucleus impacts adult neurogenesis in a rat model of Parkinson’s disease. *Neurobiol. Dis.* 42 284–291. 10.1016/j.nbd.2011.01.018 21296669

[B48] Kohl Z. Ben Abdallah N. Vogelgsang J. Tischer L. Deusser J. Amato D. (2016). Severely impaired hippocampal neurogenesis associates with an early serotonergic deficit in a BAC α-synuclein transgenic rat model of Parkinson’s disease. *Neurobiol. Dis.* 85 206–217. 10.1016/j.nbd.2015.10.021 26523794 PMC4974940

[B49] Kordower J. Olanow C. Dodiya H. Chu Y. Beach T. Adler C. (2013). Disease duration and the integrity of the nigrostriatal system in Parkinson’s disease. *Brain* 136(Pt 8), 2419–2431. 10.1093/brain/awt192 23884810 PMC3722357

[B50] Lagace D. Fischer S. Eisch A. (2007). Gender and endogenous levels of estradiol do not influence adult hippocampal neurogenesis in mice. *Hippocampus* 17 175–180. 10.1002/hipo.20265 17286277

[B51] Li L. El-Hayek Y. Liu B. Chen Y. Gomez E. Wu X. (2008). Direct-current electrical field guides neuronal stem/progenitor cell migration. *Stem Cells* 26 2193–2200. 10.1634/stemcells.2007-1022 18556511

[B52] Marinova-Mutafchieva L. Sadeghian M. Broom L. Davis J. Medhurst A. Dexter D. (2009). Relationship between microglial activation and dopaminergic neuronal loss in the substantia nigra: A time course study in a 6-hydroxydopamine model of Parkinson’s disease. *J. Neurochem.* 110 966–975. 10.1111/j.1471-4159.2009.06189.x 19549006

[B53] Marxreiter F. Nuber S. Kandasamy M. Klucken J. Aigner R. Burgmayer R. (2009). Changes in adult olfactory bulb neurogenesis in mice expressing the A30P mutant form of alpha-synuclein. *Eur. J. Neurosci.* 29 879–890. 10.1111/j.1460-9568.2009.06641.x 19291219

[B54] Mundiñano I. Caballero M. Ordóñez C. Hernandez M. DiCaudo C. Marcilla I. (2011). Increased dopaminergic cells and protein aggregates in the olfactory bulb of patients with neurodegenerative disorders. *Acta Neuropathol.* 122 61–74. 10.1007/s00401-011-0830-2 21553300

[B55] Musacchio T. Rebenstorff M. Fluri F. Brotchie J. Volkmann J. Koprich J. (2017). Subthalamic nucleus deep brain stimulation is neuroprotective in the A53T α-synuclein Parkinson’s disease rat model. *Ann. Neurol.* 81 825–836. 10.1002/ana.24947 28470693 PMC5519923

[B56] Nuber S. Harmuth F. Kohl Z. Adame A. Trejo M. Schönig K. (2013). A progressive dopaminergic phenotype associated with neurotoxic conversion of α-synuclein in BAC-transgenic rats. *Brain* 136(Pt 2), 412–432. 10.1093/brain/aws358 23413261 PMC3572936

[B57] Odekerken V. van Laar T. Staal M. Mosch A. Hoffmann C. Nijssen P. (2013). Subthalamic nucleus versus globus pallidus bilateral deep brain stimulation for advanced Parkinson’s disease (NSTAPS study): A randomised controlled trial. *Lancet Neurol.* 12 37–44. 10.1016/S1474-4422(12)70264-8 23168021

[B58] Paxinos G. Watson C. (2007). *The Rat Brain in Stereotaxic Coordinates.* Amsterdam: Academic Press.

[B59] Petry-Schmelzer J. Krause M. Dembek T. Horn A. Evans J. Ashkan K. (2019). Non-motor outcomes depend on location of neurostimulation in Parkinson’s disease. *Brain* 142 3592–3604. 10.1093/brain/awz285 31553039

[B60] Polissidis A. Koronaiou M. Kollia V. Koronaiou E. Nakos-Bimpos M. Bogiongko M. (2021). Psychosis-like behavior and hyperdopaminergic dysregulation in human α-Synuclein BAC transgenic rats. *Mov. Disord.* 36 716–728. 10.1002/mds.28383 33200461

[B61] Rodriguez-Blazquez C. Schrag A. Rizos A. Chaudhuri K. Martinez-Martin P. Weintraub D. (2020). Prevalence of non-motor symptoms and non-motor fluctuations in Parkinson’s Disease Using the MDS-NMS. *Mov Disord. Clin. Pract.* 8 231–239. 10.1002/mdc3.13122 33553493 PMC7853195

[B62] Saatçi Ö Yılmaz N. Zırh A. Yulug B. (2019). The therapeutic effect of deep brain stimulation on olfactory functions and clinical scores in Parkinson’s disease. *J. Clin. Neurosci.* 68 55–61. 10.1016/j.jocn.2019.07.055 31383472

[B63] Schmitt O. Eipert P. Kettlitz R. Leßmann F. Wree A. (2016). The connectome of the basal ganglia. *Brain Struct. Funct.* 221 753–814. 10.1007/s00429-014-0936-0 25432770

[B64] Sefton E. Iwasa S. Morrison T. Naguib H. Popovic M. Morshead C. (2020). Electric field application in vivo regulates neural precursor cell behavior in the adult mammalian forebrain. *eNeuro* 7:ENEURO.0273-20.2020. 10.1523/ENEURO.0273-20.2020 32719101 PMC7452733

[B65] Snyder J. Hong N. McDonald R. Wojtowicz J. (2005). A role for adult neurogenesis in spatial long-term memory. *Neuroscience* 130 843–852. 10.1016/j.neuroscience.2004.10.009 15652983

[B66] Somensi N. Lopes S. Gasparotto J. Mayer Gonçalves R. Tiefensee-Ribeiro C. Oppermann Peixoto D. (2021). Role of toll-like receptor 4 and sex in 6-hydroxydopamine-induced behavioral impairments and neurodegeneration in mice. *Neurochem. Int.* 151:105215. 10.1016/j.neuint.2021.105215 34710535

[B67] Spieles-Engemann A. Steece-Collier K. Behbehani M. Collier T. Wohlgenant S. Kemp C. (2011). Subthalamic nucleus stimulation increases brain derived neurotrophic factor in the nigrostriatal system and primary motor cortex. *J. Parkinsons Dis.* 1 123–136. 22328911 PMC3275429

[B68] Stone S. Teixeira C. Devito L. Zaslavsky K. Josselyn S. Lozano A. (2011). Stimulation of entorhinal cortex promotes adult neurogenesis and facilitates spatial memory. *J. Neurosci.* 31 13469–13484. 10.1523/JNEUROSCI.3100-11.2011 21940440 PMC6623309

[B69] Suzuki K. Okada K. Wakuda T. Shinmura C. Kameno Y. Iwata K. (2010). Destruction of dopaminergic neurons in the midbrain by 6-hydroxydopamine decreases hippocampal cell proliferation in rats: Reversal by fluoxetine. *PLoS One* 5:e9260. 10.1371/journal.pone.0009260 20174647 PMC2822849

[B70] Tanapat P. Hastings N. Reeves A. Gould E. (1999). Estrogen stimulates a transient increase in the number of new neurons in the dentate gyrus of the adult female rat. *J. Neurosci.* 19 5792–5801. 10.1523/JNEUROSCI.19-14-05792.1999 10407020 PMC6783062

[B71] Terreros-Roncal J. Moreno-Jiménez E. Flor-García M. Rodríguez-Moreno C. Trinchero M. Cafini F. (2021). Impact of neurodegenerative diseases on human adult hippocampal neurogenesis. *Science* 374 1106–1113. 10.1126/science.abl5163 34672693 PMC7613437

[B72] Toda H. Hamani C. Fawcett A. Hutchison W. Lozano A. (2008). The regulation of adult rodent hippocampal neurogenesis by deep brain stimulation. *J. Neurosurg.* 108 132–138. 10.3171/JNS/2008/108/01/0132 18173322

[B73] Tolosa E. Gaig C. Santamaría J. Compta Y. (2009). Diagnosis and the premotor phase of Parkinson disease. *Neurology* 72(7 Suppl.), S12–S20. 10.1212/WNL.0b013e318198db11 19221308

[B74] Tronci E. Shin E. Björklund A. Carta M. (2012). Amphetamine-induced rotation and L-DOPA-induced dyskinesia in the rat 6-OHDA model: A correlation study. *Neurosci. Res.* 73 168–172. 10.1016/j.neures.2012.03.004 22450171

[B75] van den Berge S. van Strien M. Korecka J. Dijkstra A. Sluijs J. Kooijman L. (2011). The proliferative capacity of the subventricular zone is maintained in the parkinsonian brain. *Brain* 134(Pt 11), 3249–3263. 10.1093/brain/awr256 22075520

[B76] van Praag H. Christie B. Sejnowski T. Gage F. (1999). Running enhances neurogenesis, learning, and long-term potentiation in mice. *Proc. Natl. Acad. Sci. U. S. A.* 96 13427–13431. 10.1073/pnas.96.23.13427 10557337 PMC23964

[B77] van Rienen U. (2001). *Numerical Methods in Computational Electrodynamics - Linear Systems in Practical Applications.* Berlin: Springer.

[B78] Vedam-Mai V. Gardner B. Okun M. Siebzehnrubl F. Kam M. Aponso P. (2014). Increased precursor cell proliferation after deep brain stimulation for Parkinson’s disease: A human study. *PLoS One* 9:e88770. 10.1371/journal.pone.0088770 24594681 PMC3940428

[B79] Vega C. Peterson D. (2005). Stem cell proliferative history in tissue revealed by temporal halogenated thymidine analog discrimination. *Nat. Methods* 2 167–169. 10.1038/nmeth741 15782184

[B80] Walsh S. Finn D. Dowd E. (2011). Time-course of nigrostriatal neurodegeneration and neuroinflammation in the 6-hydroxydopamine-induced axonal and terminal lesion models of Parkinson’s disease in the rat. *Neuroscience* 175 251–261. 10.1016/j.neuroscience.2010.12.005 21145947

[B81] Winner B. Desplats P. Hagl C. Klucken J. Aigner R. Ploetz S. (2009). Dopamine receptor activation promotes adult neurogenesis in an acute Parkinson model. *Exp. Neurol.* 219 543–552. 10.1016/j.expneurol.2009.07.013 19619535 PMC5038985

[B82] Winner B. Melrose H. Zhao C. Hinkle K. Yue M. Kent C. (2011). Adult neurogenesis and neurite outgrowth are impaired in LRRK2 G2019S mice. *Neurobiol. Dis.* 41 706–716. 10.1016/j.nbd.2010.12.008 21168496 PMC3059106

[B83] Winner B. Rockenstein E. Lie D. Aigner R. Mante M. Bogdahn U. (2008). Mutant alpha-synuclein exacerbates age-related decrease of neurogenesis. *Neurobiol. Aging* 29 913–925. 10.1016/j.neurobiolaging.2006.12.016 17275140 PMC2896275

[B84] Winocur G. Wojtowicz J. Sekeres M. Snyder J. Wang S. (2006). Inhibition of neurogenesis interferes with hippocampus-dependent memory function. *Hippocampus* 16 296–304. 10.1002/hipo.20163 16411241

[B85] Wolz M. Hauschild J. Koy J. Fauser M. Klingelhöfer L. Schackert G. (2012). Immediate effects of deep brain stimulation of the subthalamic nucleus on nonmotor symptoms in Parkinson’s disease. *Parkinsonism Relat. Disord.* 18 994–997. 10.1016/j.parkreldis.2012.05.011 22682974

[B86] Yagi S. Galea L. (2019). Sex differences in hippocampal cognition and neurogenesis. *Neuropsychopharmacology* 44 200–213. 10.1038/s41386-018-0208-4 30214058 PMC6235970

[B87] Zhou H. Zhu J. Jia J. Xiang W. Peng H. Zhang Y. (2022). The antidepressant effect of nucleus accumbens deep brain stimulation is mediated by parvalbumin-positive interneurons in the dorsal dentate gyrus. *Neurobiol. Stress* 21:100492. 10.1016/j.ynstr.2022.100492 36532368 PMC9755020

