# Supplementary material for: Subthalamic nucleus but not entopeduncular nucleus deep brain stimulation enhances neurogenesis in the SVZ-olfactory bulb system of Parkinsonian rats
Source: Front Cell Neurosci. 2024 Apr 30;18:1396780. doi: 10.3389/fncel.2024.1396780 (PMC11091264; doi:10.3389/fncel.2024.1396780)
Supplement: Supplementary file 1 [file Table_1.DOCX]

Supplementary Material

# Supplementary Methods

# *6-OHDA lesioning.* All rats underwent right-sided unilateral 6-hydroxydopamine (6-OHDA; Sigma-Aldrich, Taufkirchen, Germany) lesioning (3.6 mg/ml 0.9%NaCl with 0.2 mg/ml ascorbic acid) to generate a reliable dopaminergic degeneration in the ventral midbrain as described previously in detail [1]. Briefly, rats were anesthetised with isoflurane followed by weight-adapted intraperitoneal injections of ketamine/xylazine and injected with 6-OHDA into the right median forebrain bundle according to the rat brain atlas at the following coordinates: 3 µl at tooth bar (TB) -2.3; anterior-posterior (AP) -4.4; medial-lateral (ML) -1.2; dorsal-ventral (DV) -7.8 and 2.5 µl at TB +3.4; AP -4.0; ML -0.8; DV -8.0 from bregma and dura, respectively [2] using a stereotactic frame (Stoelting Neuroscience, Dublin, Ireland). Successful lesioning was quantified by amphetamine-induced rotational behaviour (5 mg/kg body weight (BW) D-amphetamine hemisulfate, Sigma-Aldrich, Dorset, UK; cohort 1; > 9 rpm over 60 min). All animals were divided into closely matched groups according to their quantitative rotational behaviour: STN_SHAM_ animals displayed an average amphetamine-induced rotational behaviour of 17.4±2.7 turns/min in contrast to STN_STIM_ animals with 17.4±2.0 turns/min; EPN_SHAM_ animals showed 13.8±1.7 turns/min and EPN_STIM_ rats 16.2±1.2 turns/min (F(3, 18)=0.83; P=0.49, from one-way ANOVA; published as supplementary figure S1 in (Fauser et al., 2021)).

# *DBS Surgery.* For our long-term DBS study, we implanted commercially available unipolar platinum-iridium electrodes (Plastics One, TX, USA) bilaterally into either the STN at the following coordinates: AP -3.5 mm, ML ±2.4 mm, DV -7.6 mm, all from bregma and dura or into the EPN (homologue of human internal globus pallidus; coordinates: AP -2.4 mm; ML ±2.9 mm, DV -7.5 mm) of all animals. After fixation to the skull with dental cement (Technovit®, Heraeus Kulzer, Hanau, Germany), electrodes were connected to a six-channel pedestal (P1 Technologies, Roanoke, VA, USA). Rats were left to recover for at least seven days until either DBS onset or sham stimulation; see Fig. 1a for experimental setups). We used external high-frequency stimulators carried in a rodent backpack with the following stimulation parameters: 200 µA, 130 Hz and 90 µs continuously for five weeks [3, 4]. Sham-stimulated animals received identical treatments, including dummy stimulators in their backpacks. At the end of the DBS period, stimulators were turned off and animals were kept for another seven days. They were then anesthetised and transcardially perfused with 4% paraformaldehyde (PFA). Brains were harvested and immersed in 4% PFA for an additional 24 hours, dehydrated in 30% sucrose, snap-frozen and stored at -80°C until further processing. Electrode placement within the respective target regions was histologically confirmed for the herein reported animals as already published in [1].

# *Immunohistochemistry.* For triple immunostaining, we used 40 µm free-floating coronal brain sections were rinsed with 1x tris-buffered saline (TBS), permeabilised in 2 N HCl at 37°C for 20 min, rinsed with TBS again and transferred to blocking solution (8 % donkey serum, 2 % Triton-X in 1x TBS) for 30 min. Sections were then incubated with the primary antibodies at 4°C overnight . The following day, sections were washed in 1xTBS, blocked and incubated with the following secondary antibodies at room temperature for 1 h: Alexa488-conjugated anti-rat IgG, Alexa594-conjugated anti-mouse IgG, Alexa647-conjugated anti-rabbit IgG (all 1:500, all from Molecular Probes, OR, US), Alexa647-conjugated anti-chicken IgG (1:400; Dianova, Hamburg, Germany). Cell nuclei were counterstained using bisbenzimide H33342 fluorochrome trihydrochloride (Invitrogen, CA, US) or 4′, 6-diamidino-2-phenylindole dihydrochloride (DAPI; Sigma-Aldrich, Taufkirchen, Germany) and mounted with Vectashield Mounting Medium (Vector Laboratories, CA, US) or Fluoromount-G (Southern Biotech, AL, US).

# Supplementary Materials

- **Figure S1.** Effects of deep brain stimulation in the subthalamic nucleus (STN-DBS) or the entopeduncular nucleus (EPN-DBS) on adult neurogenesis in the olfactory bulb (OB) in non-lesioned hemispheres of Parkinsonian rats.
- **Figure S2.** Effects of deep brain stimulation in the subthalamic nucleus (STN-DBS) and entopeduncular nucleus (EPN) on adult neurogenesis in the dentate gyrus (DG) of hemiparkinsonian rats.
- **Figure S3.** Deep brain stimulation in the subthalamic nucleus (STN-DBS) does not persistently affect adult neural stem/progenitor cell (aNSC) proliferation in the subventricular zone (SVZ) and rostral migratory system (RMS) of hemiparkinsonian rats.
- **Figure S4.** Distribution of the electric fields induced by STN-DBS and EPN-DBS, sagittal view.
- **Table S1.** Comparison of effects of persistent 6-OHDA lesion on the proliferation of adult neural stem cells in the olfactory bulb and dentate gyrus.
- **Table S2.** Comparison of effects of DBS on the proliferation of adult neural stem cells in the olfactory bulb and dentate gyrus in non-lesioned hemispheres.
- **Video S1 (separate file).** The volume of tissue activated induced by deep brain stimulation of the subthalamic nucleus (STN-DBS).
- **Video S2 (separate file).** The volume of tissue activated induced by deep brain stimulation of the entopeduncular nucleus (EPN-DBS).

# Supplementary Figures

**Supplementary Figure S1**


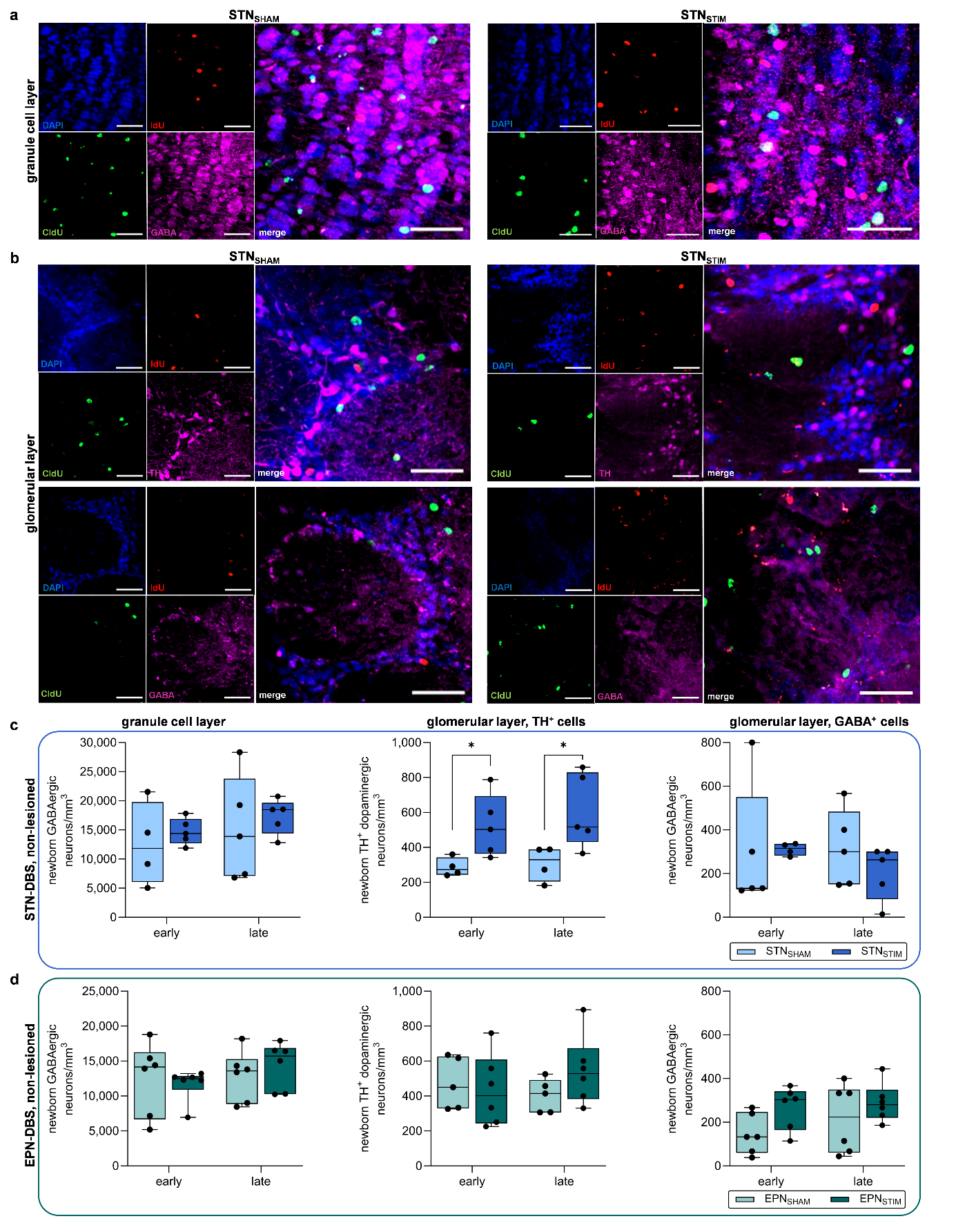


**Supplementary Figure S1. Effects of deep brain stimulation in the subthalamic nucleus (STN-DBS) or the entopeduncular nucleus (EPN-DBS) on adult neurogenesis in the olfactory bulb (OB) in non-lesioned hemispheres of hemiparkinsonian rats.**

**(a, b)** Representative immunohistological images of newborn neurons in the OB in STN_SHAM_ and STN_STIM_ conditions. IdU (red) labels proliferating neurons that were generated early (two days) after DBS initiation, while CldU (green) indicates newborn neurons after three weeks of continuous DBS. GABAergic neurons (pink) were found both in the granule cell layer **(a)** and in the glomerular layer **(b, lower panel)**, while TH^+^ dopaminergic neurons (pink) were restricted to the latter **(b, upper panel)**. Cell nuclei were counterstained with DAPI. Scale bar, 50 µm. **(c)** Bilateral STN-DBS did not alter the numbers of newborn GABAergic neurons in the granule cell layer or glomerular layer, while dopaminergic neuron counts in the glomerular layer were significantly increased early after STN-DBS onset and three weeks later in the non-lesioned hemisphere of hemiparkinsonian rats compared to sham stimulation. **(d)** Bilateral EPN-DBS did neither alter the numbers of newborn GABAergic neurons in the granule cell layer or glomerular layer, nor dopaminergic neuron counts in the glomerular layer early after EPN-DBS onset and three weeks later in the non-lesioned hemisphere of hemiparkinsonian rats compared to sham stimulation. Data are presented as boxplots with a central mark at the median, bottom, and top edges of the boxes at 25^th^ and 75^th^ percentiles, respectively, and whiskers at the minimum/maximum (dots represent individual values). * represents *P*<0.05 from Mann-Whitney-U-tests.

Abbreviations: DBS – deep brain stimulation; STN – subthalamic nucleus; OB – olfactory bulb; IdU – 5-Iodo-2′-deoxyuridine; CldU - 5-chloro-2'-deoxyuridine; GABA – gamma aminobutyric acid; TH – tyrosine hydroxylase; DAPI - 4′,6-diamidino-2-phenyl-indol-dihydrochloride.

**Supplementary Figure S2**


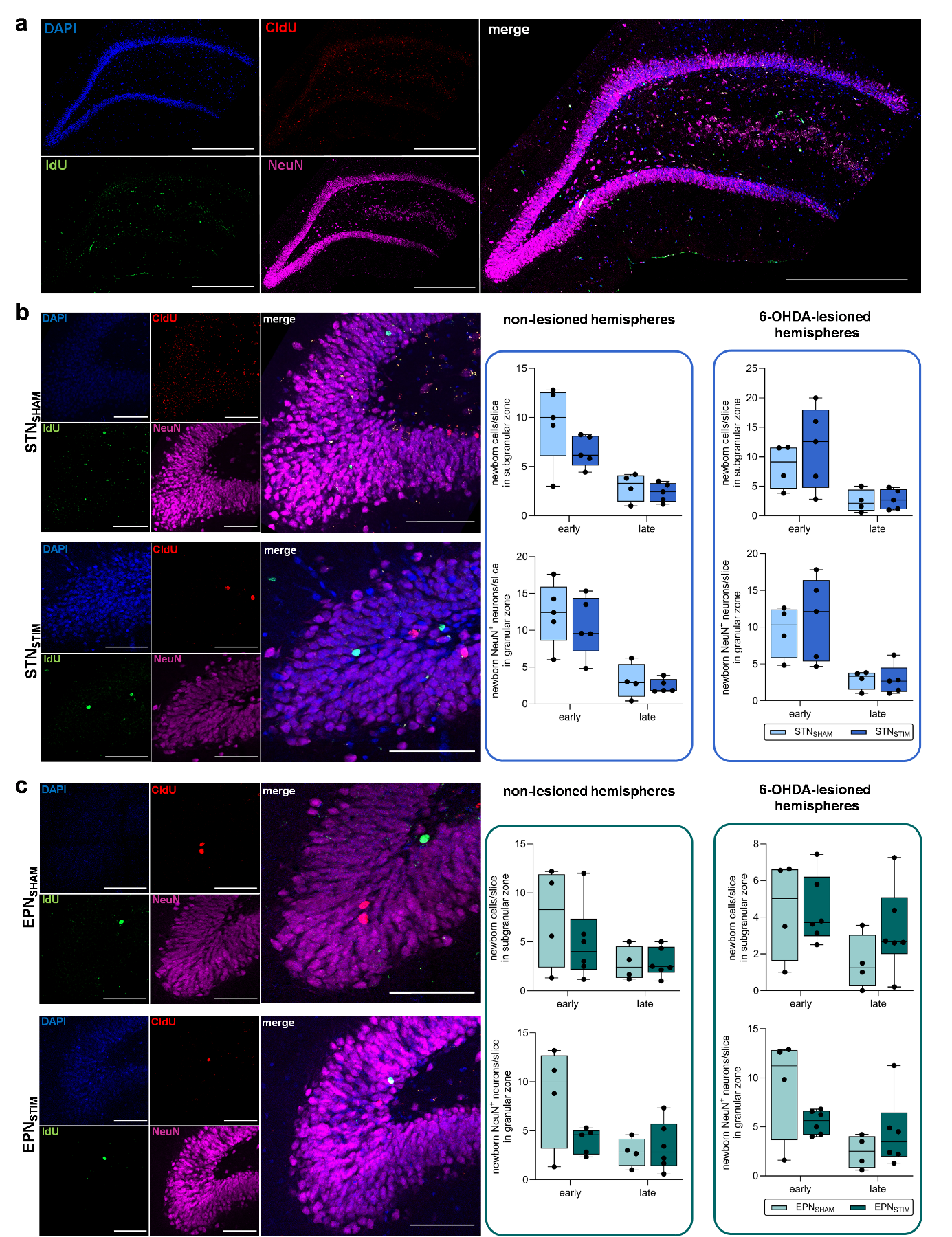


**Supplementary Figure S2.** **Effects of deep brain stimulation in the subthalamic nucleus (STN-DBS) and entopeduncular nucleus (EPN-DBS) on adult neurogenesis in the dentate gyrus (DG) of hemiparkinsonian rats.**

**(a)** Representative immunohistological overview of the DG with newborn neurons (red; green). Neurons are counterstained with NeuN (pink), while cell nuclei are labelled with DAPI (blue). Scale bar, 500 µm. **(b)** Left panel shows representative immunohistological images of newborn neurons in the DG in STN_SHAM_ and STN_STIM_ conditions. IdU (red) labels newborn neurons that were generated early (two days) after DBS onset, while CldU (green) indicates newborn neurons after three weeks of continuous DBS. Cell nuclei were counterstained with DAPI. Scale bar, 100 µm. Quantitative immunohistological analyses (right panels) revealed that bilateral STN-DBS did not alter the numbers of newborn neurons neither in the subgranular zone (upper panel) nor in the granular zone (lower panel) early after DBS onset and three weeks later compared to sham stimulation in both hemispheres. **(c)** Left panel shows representative immunohistological images of newborn neurons in the DG in EPN_SHAM_ and EPN_STIM_ conditions. IdU (red) labels newborn neurons that were generated early (two days) after DBS onset, while CldU (green) indicates newborn neurons after three weeks of continuous DBS. Cell nuclei were counterstained with DAPI. Scale bar, 100 µm. Quantitative analyses (right panels) showed that bilateral EPN-DBS did not alter the numbers of newborn neurons neither in the subgranular zone (upper panel) nor in the granular zone (lower panel) early after DBS onset and three weeks later compared to sham stimulation in both hemispheres. Data are presented as boxplots with a central mark at the median, bottom, and top edges of the boxes at 25^th^ and 75^th^ percentiles, respectively, and whiskers the minimum/maximum (dots represent individual values). No significant group differences were detected (Mann-Whitney-U-tests).

Abbreviations: DBS – deep brain stimulation; STN – subthalamic nucelus; EPN – entopeduncular nucleus; 6-OHDA – 6-hydroxydopamine; IdU – 5-Iodo-2′-deoxyuridine; CldU - 5-chloro-2'-deoxyuridine; DAPI - 4′,6-diamidino-2-phenyl-indol-dihydrochloride.

**Supplementary Figure S3**


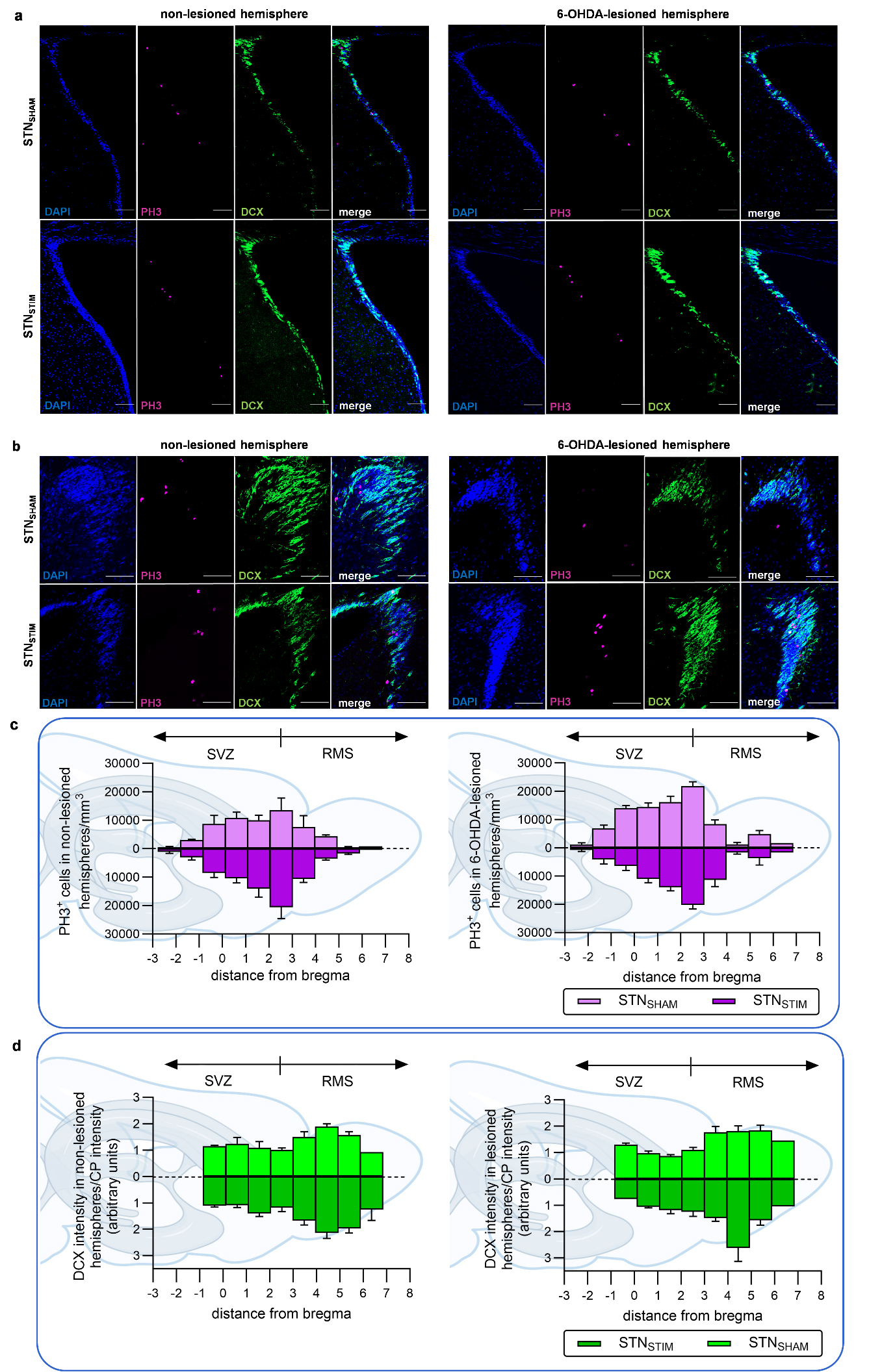


**Supplementary Figure S3.** **Deep brain stimulation in the subthalamic nucleus (STN-DBS) does not persistently affect adult neural stem/progenitor cell (aNSC) proliferation in the subventricular zone (SVZ) and rostral migratory system (RMS) of hemiparkinsonian rats.**

**(a**) Representative immunohistological images of the neurogenic niche of the SVZ of the lateral ventricles. Proliferating stem cells were labelled with PH3 (pink), while neuroblasts were DCX^+^ (green). Cell nuclei were counterstained with DAPI. Scale bars, 100 µm. **(b)** Representative immunohistological images of the RMS with proliferating PH3^+^ cells and DCX^+^ neuroblasts in STN_SHAM_ and STN_STIM_ conditions one week after termination of five weeks of continuous DBS. Cell nuclei were counterstained with DAPI. Scale bars, 100 µm. **(c)** Neural stem and progenitor cell proliferation along the ventricular neuraxis relative to bregma in hemiparkinsonian rats on the non-lesioned (left panel) and lesioned (right panel) hemispheres. Upper histograms depict PH3^+^ proliferating aNSCs in STN_SHAM_ condition, while lower histograms represent proliferating aNSCs in STN_STIM_ animals one week after termination of chronic DBS over five weeks. We found no persistent effect on aNSC proliferation after termination of STN-DBS compared to sham stimulation. **(d)** DCX intensity measurements along the ventricular neuraxis relative to bregma in hemiparkinsonian rats on the non-lesioned (left panel) and lesioned (right panel) hemispheres. Upper histograms depict DCX intensity relative to a reference region within the caudate putamen as an indicator of neuroblast density in STN_SHAM_ condition, while lower histograms represent STN_STIM_ animals one week after termination of chronic DBS over five weeks. There was no persistent effect on DCX^+^ neuroblast density in the SVZ and along the RMS after termination of STN-DBS compared to sham stimulation. Data are presented as mean values ± SEM. No significant group differences between stimulated and non-stimulated animals were detected when data where compared at a sum level (Mann-Whitney-U-tests).

Abbreviations: DBS – deep brain stimulation; STN – subthalamic nucleus; aNSC – adult neural stem/progenitor cell; SVZ – subventricular zone; PH3 – phospho-histone H3; DCX – doublecortin X; RMS – rostral migratory system.

**Supplementary Figure S4**


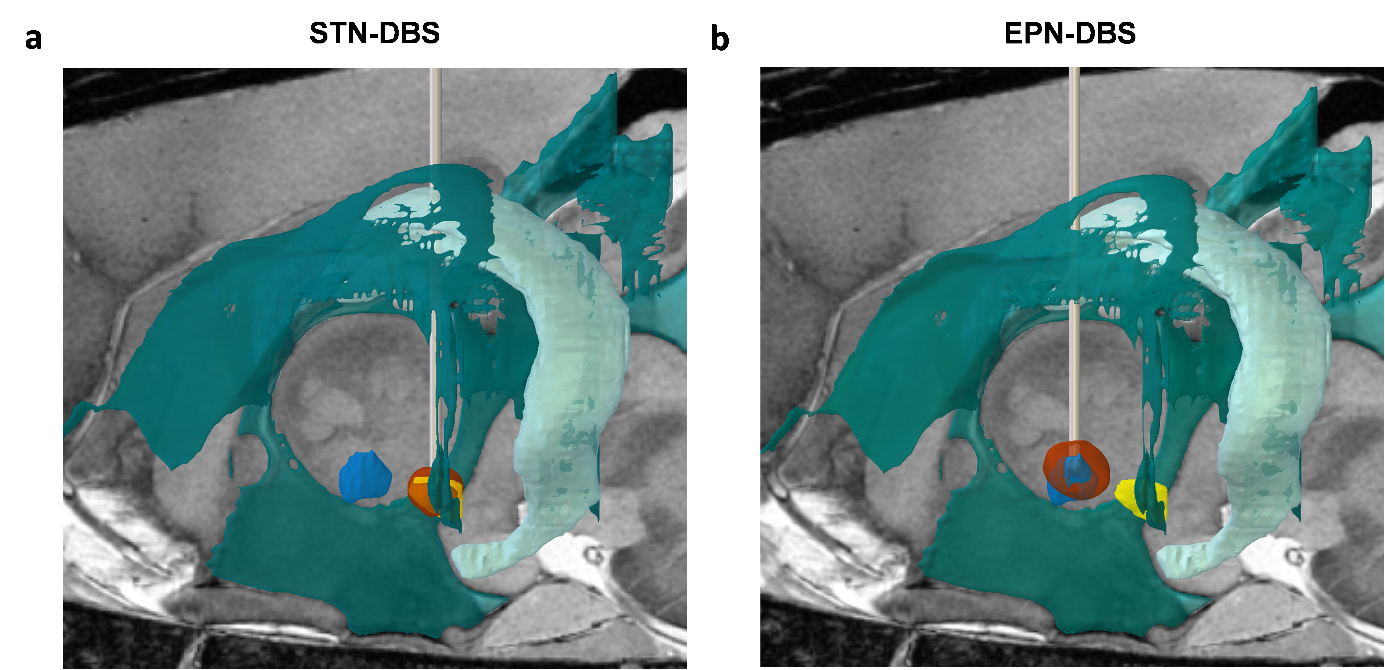


**Supplementary Figure S4.** **Distribution of the electric fields induced by STN-DBS and EPN-DBS, sagittal view**.

**(a,b)** Additional electrode reconstruction into the **(a)** STN (yellow structure) and **(b)** EPN (blue structure) in sagittal views illustrating the volume of tissue activated (VTA, circumferential red circle around the electrodes) with respect to the two neurogenic niches, namely the SVZ adjusted to the lateral ventricles of the ventricular system (dark green structure) and the dentate gyrus (bright green structure).

Abbreviations: STN – subthalamic nucleus; EPN – entopeduncular nucleus; DBS – deep bran stimulation; VTA – volume of tissue activated; SVZ – subventricular zone.

**Supplementary Table S1.** Comparison of effects of persistent 6-OHDA lesion on the proliferation of adult neural stem cells in the olfactory bulb and dentate gyrus in sham-treated animals.

|  | **STN** | | |  | **EPN** | | |
| --- | --- | --- | --- | --- | --- | --- | --- |
|  | **Healthy, non-lesioned side** | **Parkinsonian,**  **6-OHDA-lesioned side** | ***P* values** |  | **Healthy, non-lesioned side** | **Parkinsonian,**  **6-OHDA-lesioned side** | ***P* values** |
| **Olfactory bulb** (per mm^3^) |  | | |  |  | | |
| GCL_early_ | 12,291±2,777 | 10,186±1,106 | *P*=0.54 |  | 12,480±2,125 | 13,545±577 | *P*=0.23 |
| GCL_late_ | 15,136±4,013 | 12,955±1,336 | *P*=0.60 |  | 12,860±1,486 | 14,907±1224 | *P*=0.81 |
| GL_early_ – TH^+^ | 286±27 | 249±22 | *P*=0.63 |  | 472±67 | 261±21 | *P*=0.18 |
| GL_late_ – TH^+^ | 307±50 | 218±31 | *P*=0.27 |  | 402±43 | 364±139 | *P*=0.78 |
| GL_early_ – GABA^+^ | 298±130 | 313±83 | *P*=0.87 |  | 146±37 | 292±52 | *P*=0.07 |
| GLlate – GABA^+^ | 314±79 | 286±64 | *P*=0.84 |  | 215±64 | 295±103 | *P*=0.54 |
| **Dentate gyrus** (per slice) | | | |  |  | | |
| SGZ_early_ | 9.5±1.8 | 8.4±1.9 | *P*=0.66 |  | 7.5±2.5 | 4.4±1.4 | *P*=0.46 |
| SGZ_late_ | 2.9±0.7 | 2.5±0.9 | *P*=0.63 |  | 2.8±0.9 | 1.5±0.7 | *P*=0.40 |
| GZ_early_ | 12.3±1.9 | 9.5±1.8 | *P*=0.42 |  | 8.6±2.6 | 9.2±2.6 | *P*=0.90 |
| GZ_late_ | 3.1±1.2 | 2.9±0.7 | *P*=0.67 |  | 2.8±0.7 | 2.5±0.8 | *P*=0.78 |

Data are presented as mean ± SEM (n=4-6) and normalized to respective tissue volumes (in mm^3^; for OB) or to numbers of quantified slices (for DG). *P*-values are from Wilcoxon matched-pairs signed rank test to determine mean differences between groups. Abbreviations: STN – subthalamic nucleus; EPN – entopeduncular nucleus; 6-OHDA – 6-hydroxydopamine; GCL – granule cell layer; GL – glomerular layer; TH -tyrosine hydroxylase; GABA – gamma amino butyric acid; SGZ – subgranular zone; GZ – granular zone.

**Supplementary Table S2.** Comparison of effects of DBS on the proliferation of adult neural stem cells in the olfactory bulb and dentate gyrus in non-lesioned hemispheres.

|  | **STN** | | |  | **EPN** | | |
| --- | --- | --- | --- | --- | --- | --- | --- |
|  | **SHAM** | **STIM** | ***P* values** |  | **SHAM** | **STIM** | ***P* values** |
| **Olfactory bulb** (per mm^3^) |  | | |  |  | | |
| GCL_early_ | 12,291±2777 | 14,710±1,022 | *P*=0.44 |  | 12,480±2,125 | 11,685±958 | *P*=0.74 |
| GCL_late_ | 15,136±4013 | 17,326±1,356 | *P*=0.62 |  | 12,860±1,486 | 14,404±1360 | *P*=0.46 |
| GL_early_ – TH^+^ | 286±27 | 524±80 | *P*=0.03 |  | 472±67 | 433±84 | *P*=0.73 |
| GL_late_ – TH^+^ | 307±50 | 608±95 | *P=*0.04 |  | 402±43 | 547±80 | *P*=0.17 |
| GL_early_ – GABA^+^ | 298±130 | 311±14 | *P*=0.27 |  | 146±37 | 267±40 | *P*=0.15 |
| GL_late_ – GABA^+^ | 314±79 | 254±35 | *P*=0.55 |  | 215±64 | 290±36 | *P*=0.34 |
| **Dentate gyrus** (per number of slices) | | | |  |  | | |
| SGZ_early_ | 9.5±1.8 | 6.5±0.7 | *P*=0.16 |  | 7.5±2.5 | 4.9±1.6 | *P*=0.38 |
| SGZ_late_ | 2.9±0.7 | 2.4±0.4 | *P*=0.50 |  | 2.8±0.9 | 2.9±0.6 | *P*=0.90 |
| GZ_early_ | 12.3±1.9 | 10.5±1.8 | *P*=0.53 |  | 8.6±2.6 | 4.0±0.6 | *P*=0.09 |
| GZ_late_ | 3.1±1.2 | 2.4±0.4 | *P*=0.58 |  | 2.8±0.7 | 3.4±1.0 | *P*=0.69 |

Data are presented as mean ± SEM (n=4-6) and normalized to respective tissue volumes (in mm^3^; for SVZ and OB) or to numbers of quantified slices (for DG). Levene Test was conducted for analysis of variance; normal distribution of data was analysed via visual inspection of boxplots and Shapiro-Wilk Test. *P*-values are from unpaired two-sided t-test or Mann-Whitney-U-Test, as appropriate to determine mean differences between groups. **Abbreviations:** STN – subthalamic nucleus; EPN – entopeduncular nucleus; GCL – granule cell layer; PGL – glomerular layer; TH – tyrosine hydroxylase; GABA – gamma aminobutyric acid.

**Supplementary Video S1. The volume of tissue activated induced by deep brain stimulation in the subthalamic nucleus (STN-DBS)**

Video of electrode reconstruction into the STN (yellow structure) starting with a transversal view illustrating the volume of tissue activated (VTA, circumferential red circle around the electrodes) in respect to the two neurogenic niches, namely the SVZ adjacent to the lateral ventricles of the ventricular system (dark green structure) and the DG (bright green structure). The EPN (blue structure) has been added for comparison.

Abbreviations: STN – subthalamic nucleus; EPN – entopeduncular nucleus; DBS – deep brain stimulation; VTA – volume of tissue activated; SVZ – subventricular zone; DG – dentate gyrus.

**Supplementary Video S2. The volume of tissue activated induced by deep brain stimulation of the entopeduncular nucleus (EPN-DBS).**

Video of electrode reconstruction into the EPN (blue structure) starting with a transversal view illustrating the volume of tissue activated (VTA, circumferential red circle around the electrodes) in respect to the two neurogenic niches, namely the SVZ adjacent to the lateral ventricles of the ventricular system (dark green structure) and the DG (bright green structure). The STN (yellow structure) has been added for comparison.

Abbreviations: STN – subthalamic nucleus; EPN – entopeduncular nucleus; DBS – deep brain stimulation; VTA – volume of tissue activated; SVZ – subventricular zone; DG – dentate gyrus.

.

**Supplementary Literature**

Fauser, M., Ricken, M., Markert, F., Weis, N., Schmitt, O., Gimsa, J., . . . Storch, A. (2021). Subthalamic nucleus deep brain stimulation induces sustained neurorestoration in the mesolimbic dopaminergic system in a Parkinson's disease model. *Neurobiol Dis*, *156*, 105404. <https://doi.org/10.1016/j.nbd.2021.105404>

Golmohammadi, M. G., Blackmore, D. G., Large, B., Azari, H., Esfandiary, E., Paxinos, G., . . . Rietze, R. L. (2008). Comparative analysis of the frequency and distribution of stem and progenitor cells in the adult mouse brain. *Stem Cells*, *26*(4), 979-987. <https://doi.org/10.1634/stemcells.2007-0919>
